# Technological State of the Art of Electronic Mental Health Interventions for Major Depressive Disorder: Systematic Literature Review

**DOI:** 10.2196/12599

**Published:** 2020-01-20

**Authors:** Franziska Burger, Mark A Neerincx, Willem-Paul Brinkman

**Affiliations:** 1 Delft University of Technology Delft Netherlands; 2 Nederlandse Organisatie voor Toegepast Natuurwetenschappelijk Onderzoek (TNO) Department of Perceptual and Cognitive Systems Soesterberg Netherlands

**Keywords:** eHealth, major depressive disorder, technology, systematic review

## Abstract

**Background:**

Electronic mental (e-mental) health care for depression aims to overcome barriers to and limitations of face-to-face treatment. Owing to the high and growing demand for mental health care, a large number of such information and communication technology systems have been developed in recent years. Consequently, a diverse system landscape formed.

**Objective:**

This literature review aims to give an overview of this landscape of e-mental health systems for the prevention and treatment of major depressive disorder, focusing on three main research questions: (1) What types of systems exist? (2) How technologically advanced are these systems? (3) How has the system landscape evolved between 2000 and 2017?

**Methods:**

Publications eligible for inclusion described e-mental health software for the prevention or treatment of major depressive disorder. Additionally, the software had to have been evaluated with end users and developed since 2000. After screening, 270 records remained for inclusion. We constructed a taxonomy concerning software systems, their functions, how technologized these were in their realization, and how systems were evaluated, and then, we extracted this information from the included records. We define here as functions any component of the system that delivers either treatment or adherence support to the user. For this coding process, an elaborate classification hierarchy for functions was developed yielding a total of 133 systems with 2163 functions. The systems and their functions were analyzed quantitatively, with a focus on technological realization.

**Results:**

There are various types of systems. However, most are delivered on the World Wide Web (76%), and most implement cognitive behavioral therapy techniques (85%). In terms of content, systems contain twice as many treatment functions as adherence support functions, on average. Furthermore, autonomous systems, those not including human guidance, are equally as technologized and have one-third less functions than guided ones. Therefore, lack of guidance is neither compensated with additional functions nor compensated by technologizing functions to a greater degree. Although several high-tech solutions could be found, the average system falls between a purely informational system and one that allows for data entry but without automatically processing these data. Moreover, no clear increase in the technological capabilities of systems showed in the field, between 2000 and 2017, despite a marked growth in system quantity. Finally, more sophisticated systems were evaluated less often in comparative trials than less sophisticated ones (OR 0.59).

**Conclusions:**

The findings indicate that when developers create systems, there is a greater focus on implementing therapeutic treatment than adherence support. Although the field is very active, as evidenced by the growing number of systems developed per year, the technological possibilities explored are limited. In addition to allowing developers to compare their system with others, we anticipate that this review will help researchers identify opportunities in the field.

## Introduction

Between 2000 and 2017, researchers have reported more than 100 software interventions for depression in the scientific literature. Although all these systems have the same objective, they vary widely in both content and in the way the content is delivered. Taken together, they thus form a diverse landscape. But what does this landscape actually look like? The purpose of this literature review is to map the terrain by exploring the technological state of the art of electronic mental (e-mental) health interventions for depression.

The systems under study here strive to meet a globally growing need for depression care. The illness affects approximately 300 million people worldwide [1]. Its high lifetime prevalence and high disease burden are further exacerbated by additional episodes often following the first. This renders the pervasive provision of treatment and prevention means imperative. However, the World Health Organization estimates that, currently, half of those suffering from depression are receiving inadequate or no treatment [1].

Information and communication technology (ICT) may present a viable solution to the shortage. The rapid dissemination of ICT over the course of the past two decades has led researchers to explore the provision of therapeutic content on these platforms. Unlike face-to-face treatment, such support systems are scalable, easily accessible, cheap, and standardized, and they can reduce the fear of stigmatization, as they can be used in private and at one’s own convenience [2]. In addition to these benefits, numerous meta-analyses attest to the effectiveness of the interventions [3-5].

As a consequence of the high research interest, many systems have been developed to treat or prevent depression. Each system presents a unique solution. In light of this, several recent literature surveys point out that an analysis of the system landscape is in order, as there is little insight into the makeup of systems [2,6,7]. Where systems have been reviewed to date, authors have typically adopted one of two core perspectives. Syntheses with a *clinical psychology* perspective have addressed the effectiveness of different types of interventions [3,8,9]. Syntheses with a *(persuasive) technology* perspective, on the other hand, have addressed the functionality of systems, such as persuasive technology elements [7] or communication modality [10]. This systematic literature review takes the latter perspective. However, rather than studying in depth the implementation or impact of a specific type of function, it compares entire systems on their technological implementation. In doing so, e-mental health systems for depression are regarded as compositions of functions and assessed in terms of their technological realization. The support systems reported in the literature thus form the population under study. The main goal of this review is then to provide a comprehensive overview of the system landscape and its technological state. In addition, it identifies some of the challenges and opportunities for the field. However, linking the degree to which systems present high-tech solutions with clinical outcomes is outside of the scope of this review. Nevertheless, with the introduced system characterization and technological sophistication metric, a first step toward such studies is taken. From the extensive, domain-specific analysis presented here, we particularly expect researchers setting out to develop or study support systems for depression to benefit. It allows them to compare their system with those already in use and to identify underexplored aspects of these systems. To this end, the following three research questions are addressed:

1. What types of ICT systems for the treatment and prevention of depression have been developed?
2. How technologized are these systems?
3. How has the system landscape evolved between 2000 and 2017?

## Methods

### Literature Identification and Coding

In this section, we focus on the literature search and filtering as well as coding of data pertaining to the analyses in this study. A detailed account of the construction, the structure, and the information contained in the open-access, relational database that was created for this analysis can be found in the documents [11,12].

#### Identification

The exhaustive search for potentially relevant literature made use of 3 databases: Scopus, PubMed, and Web of Science. It included English language journal articles, conference papers, and theses published between 2000 and 2017, presenting primary research that was conducted with support systems for the prevention or treatment of major depressive disorder or dysthymia in adults. To ensure that systems were actually created and functional at some point, we only considered the literature that reported the results of a system evaluation with end users. Therefore, systems that only had published study protocols available at the time of the search (early 2017) did not qualify. Lists of search terms comprised words around the following concepts that were central to the research interest: *ICT, Health Condition, Purpose, Evaluation* (Multimedia Appendix 1). They were expanded with controlled vocabulary terms, where applicable. Systems met exclusion criteria if they were (1) employing technology for mediated communication, (2) targeting children, postpartum or pregnant women, caregivers of depressed patients, or patients with comorbid psychotic conditions, (3) only aiming to reduce stigma, (4) serving only as diagnostic tools or decision aids, (5) addressing only antidepressant treatment, and (6) having an otherwise too narrow scope, for example, a system developed for a single patient with a specific combination of comorbid conditions.

**Figure 1:**
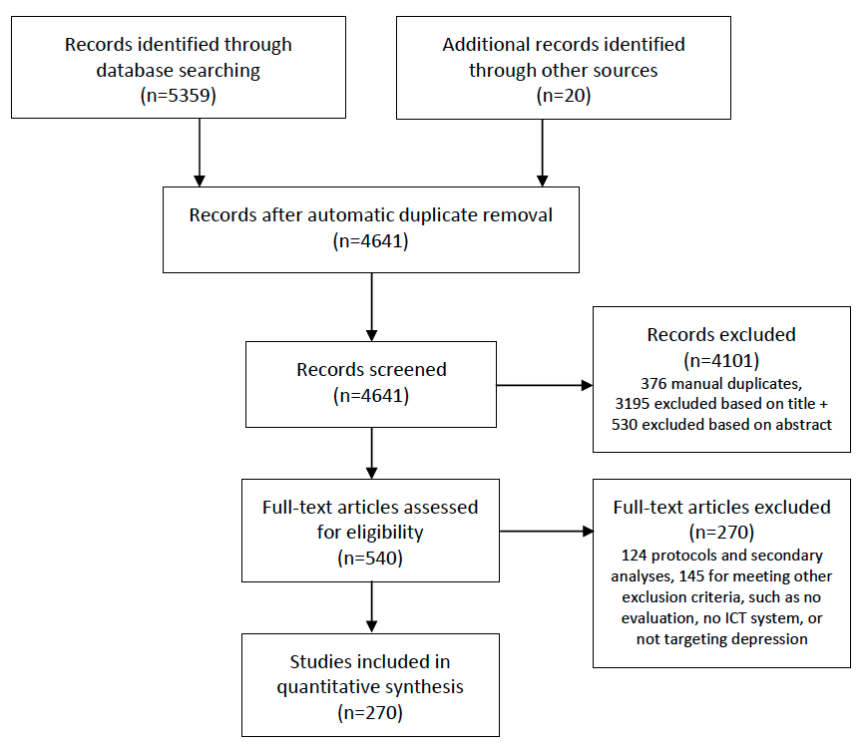
Preferred Reporting Items for Systematic Reviews and Meta-Analyses (PRISMA) diagram of the screening process, as completed by the first author. ICT: information and communication technology.

The 3 queried databases returned a total of 5359 documents. Forward and backward reference searches on previous literature reviews and meta-analyses yielded an additional 20 records. After the removal of duplicates, 4256 records remained for screening. A lenient inclusion protocol at the title and abstract stages allowed for the inclusion of as many articles as possible concerning a system. Therefore, the exclusion of articles describing study protocols and secondary analyses only occurred at the full paper screening, but they were kept as additional references for clarification purposes. The first author, with a cognitive science background, screened all records at the title, abstract, and full-text stages (see PRISMA [13] diagram in Figure 1). A second, independent coder, with a computer science background, double coded a random selection at each stage. Intercoder agreement ranged from 80% to 84%, with moderate-to-substantial intercoder reliability (Cohen kappa between 0.50 and 0.69). Multimedia Appendix 2 includes a complete list of all 270 articles included in the final synthesis.

#### Coding

To provide an overview of the aspects of software systems for depression considered here, a simplified taxonomy is presented in Multimedia Appendix 3. The extraction of information resulted in 45 coded attributes. These were either low-inference attributes, that is, the information could be directly copied from the paper, or high-inference attributes, that is, the coder needed to make inferences to arrive at the information. Second coders were neither used to refine the coding procedure nor to obtain a more reliable dataset. However, second coders did double code samples of the high-inference attributes to assess the reliability of the first coder, and the intercoder reliability measures reported here are to be regarded as an indication thereof. All analyses are based on the coding of the first author only.

**Figure 2:**
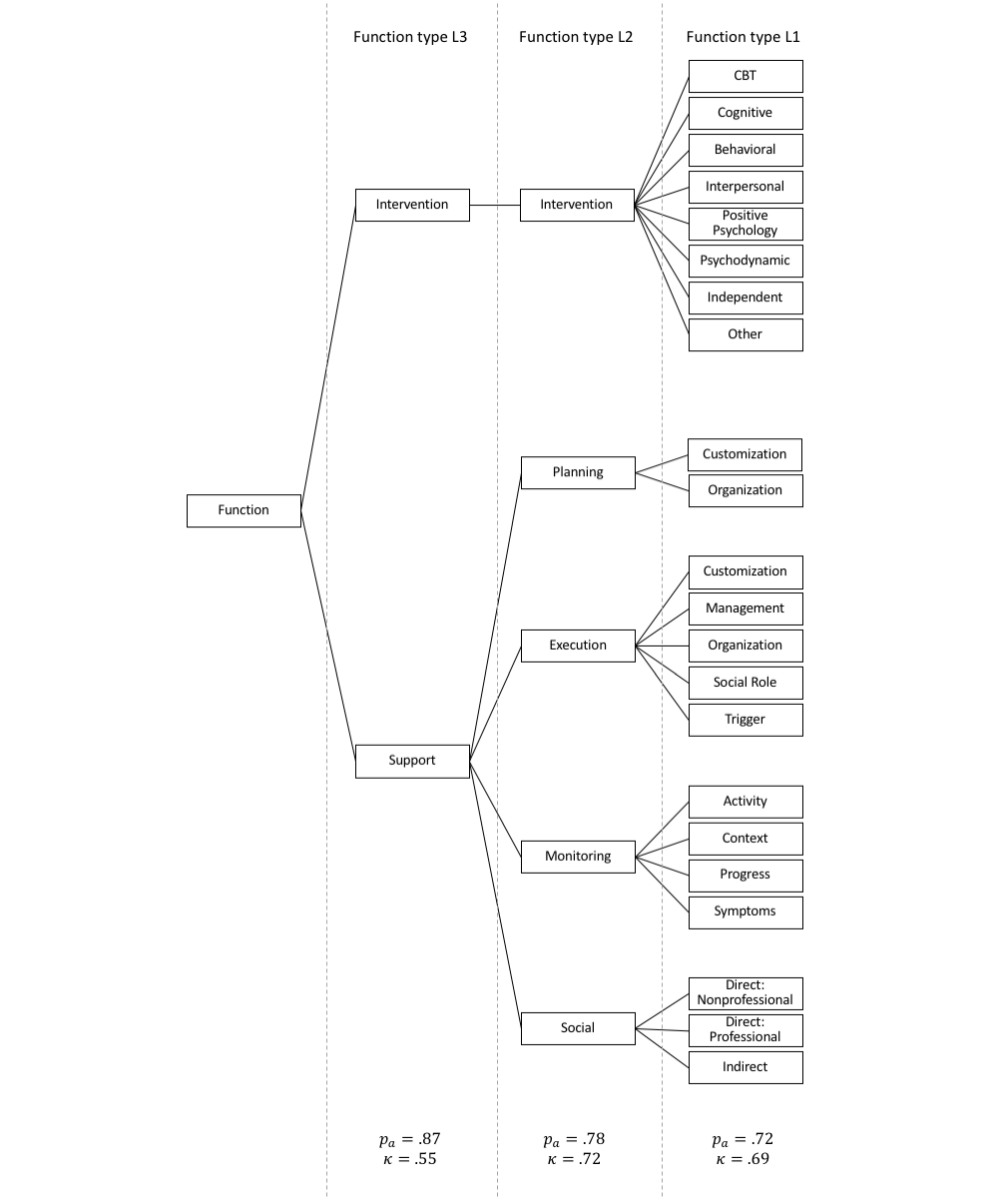
The top three levels of the function classification tree as well as the percent agreement and Cohen’s kappa for the function classification task at each of the levels. Level 0 of the tree is specified in Multimedia Appendix 2 (support functions) and Multimedia Appendix 3 (intervention functions). CBT: cognitive behavioral therapy.

A key task in the coding process was the division of systems into elementary functional parts, that is, functions. Herein, the focus was limited to functions pertaining to the higher-level layers of software architecture. For example, in the layered software architecture described in the Microsoft Application Architecture Guide [14], the functions would be located in the presentation and application layers. Cross-cutting concerns, such as security, were not considered. Additional criteria by which to evaluate software quality, for example maintainability, integration with other software, or software reliability, are also beyond the scope of this work. The construction of a classification hierarchy (Figure 2) preceded the coding process. At the fourth and highest level, two types of functions are possible: *intervention* functions, which aim to reduce depressive symptomatology in users, and *support* functions, which aim to increase adherence of the user to the intervention. An example of an intervention function would be the positive psychology exercise to count one’s blessings every night, whereas an example of a support function would be to send text message reminders to encourage the user to engage with the system. At the third level, support functions further split into helping the user in (1) planning the intervention, (2) executing the intervention, (3) self-monitoring, or (4) connecting with other supportive people. A total of 2 more refined classification levels follow. At the lowest level, 41 classifications make up the support functions (Multimedia Appendix 4) and 145 classifications make up the intervention functions (Multimedia Appendix 5). Inspiration for the lowest-level support functions came largely from persuasive technology design frameworks [15-18], whereas therapy manuals (eg, [19]) inspired the lowest-level intervention functions. These are often linked to therapeutic intervention frameworks, for example, Activity Planning is a technique of Behavioral Therapy. The intervention frameworks finally cluster into 8 *therapies* (Multimedia Appendix 5).

A second coder with a background in clinical psychology double coded two parts of the function identification task. The first part required spotting functions in the system description. Taking the functions that were found by the first coder as ground truth, interrater reliability was moderate on this part (ϕ=0.54, with a specificity [20] of *d*'=2.31). The second part required labeling snippets of text that the first coder had identified as functions. For this part, interrater reliability on the 4 different function classification levels (Figure 2) was good on average (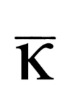
=0.63), ranging from moderate (κ=0.55) to good (κ=0.72).

Another key coding task concerned rating the degree to which each function was technologized. A set of scales, the e-mental Health Degree of Technological Sophistication (eHDTS) rating scales (Multimedia Appendix 6), were developed specifically for this task. They include one scale for intervention functions and four separate scales for each of the four types of support functions. Conceptually, the scales range from *offline* to *responsive on content* (Table 1). Although the emphasis in the interpretation of the eHDTS scales throughout this work is placed on the *interactivity* aspect, the actual scales are broader, also covering aspects such as responsiveness, personalization, data analysis, and data presentation. From here on, when directly describing the technological realization of systems or functions as measured by the scale, we refer to it as *technological sophistication*. In coding, a conservative approach ensured that the lower degree was assigned in case of doubt. Reliability levels were acceptable, with a mean correlation of 0.66 between coders. Furthermore, concurrent validity of the scales was supported by on-average moderate correlations (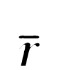
=0.53) between ratings on these scales and ratings on an unlabeled ordinal scale, that is, leaving it open to coders to decide what the different levels of technological sophistication entail.

Finally, one coder was provided with a list of function descriptions from all function types, without the function type label, and asked to assign a rating of technological sophistication to these on an unlabeled ordinal scale from 0 to 5 (uninformed). After two weeks, he was again invited to code the same functions with the appropriate scale for each function and each scale level defined (informed). The correlation between the uninformed and the informed rating (*r*=0.47) provided some indication that, although each function type had its own eHDTS scale, the five scales were sufficiently similar to allow for aggregation and cautious comparisons on a system level.

Three low-inference attributes coded were the system version, the system build year, and the evaluation quality. A *version* was defined as a modification of the system offering different functionality. For example, Lemma et al created a version with human support and a version without it [21], whereas Currie et al offer different versions to support female or male patients by providing extra content for women [22]. However, a system with an adaptive user interface based on gender was not regarded as two versions; it was regarded as one with a tailoring function. The system *build year* denotes the year in which systems were finalized, that is, the earliest year of operation mentioned in the earliest publication on the earliest version (versions and systems are simply referred to as *systems* or *software* for legibility from here on. Most analyses to follow were conducted on the body of versions rather than systems. It is made explicit when this is not the case). Finally, the *evaluation quality* received a binominal coding of high and low. A high quality meant that the system was evaluated in a comparative trial, whereas a low quality meant that it was evaluated in a single-group trial. Comparative trials encompassed randomized controlled trials, randomized comparative trials, and nonrandomized comparative trials.

**Table 1:** The degrees of the e-mental health degree of technological sophistication rating scale, abstracted over the 5 different instantiations of this scale. A diary function serves as a hypothetical example. It should be noted that this is an abstract summary of the levels across several scales. It therefore does not capture the entire technological breadth of the different scales.

Degree	Definition	Example
0: Offline	The function is not provided through the system at all or is fully carried out by a human.	Diary sent by postal mail
1: Informational	The function is provided in an informational manner.	Diary can be downloaded as PDF
2: Data entry	The function is provided in an interactive manner but without processing of input from the user.	Diary can be filled on the Web and saved
3: Form response	The function is provided in an interactive manner with processing of meta-information	Web-based diary that responds to the duration of typing
4: Content response	The function is provided in an interactive manner with processing of the content of user input.	Web-based diary that responds to the sentiment of text, which the user has written, for example, “It appears that this was a very negative experience for you.”

### Statistical Analysis

We conducted quantitative analyses with R version 3.5. All data and the full analysis script are permanently stored for public access on a national database for research data with the 4TU Center for Research Data in the Netherlands [23]. Where distributions deviated markedly from normality, nonparametric tests were used. Furthermore, we report 2 estimated *R^2^* effect size measures where *R^2^* cannot be calculated exactly. For logistic regression models, Nagelkerke pseudo *R^2^* [24] was chosen, whereas, for multilevel models, the Level1 *R^2^*, as proposed by Snijders and Bosker [25], was computed. When used, these are indicated as Nagelkerke *R^2^* and Level1 *R^2^*, respectively.

#### Characterization

To characterize systems, we regarded their composition in terms of functions and how systems differed depending on such factors as guidance or system purpose, that is, prevention or treatment. A Wilcoxon rank sum test compared the number of intervention functions with the number of support functions per system. In addition, two logistic regression models were fit. One determined whether a certain system purpose was more commonly occurring within a certain therapy type. The other tested whether autonomous or guided systems are represented to different degrees depending on purpose. Systems that include human guidance naturally have more functions, as guidance needs to be facilitated by the system somehow. This takes place by way of the direct social support functions. Thus, to allow for a fair comparison of the number of functions in autonomous versus guided systems, direct social support functions were excluded for the following three analyses. First, a linear regression examined the relationship between guidance and the number of functions of a system. Second, two more detailed analyses in the form of Wilcoxon rank sum tests considered this relationship separately for intervention and support functions.

#### Technological Sophistication

Technological sophistication was compared among the different types of functions, different types of systems, and different evaluation qualities. A correlation assessed the relationship between system size and technological sophistication, and linear regression models gave insight into the link between technological sophistication on the one hand and evaluation quality, guidance, or system purpose on the other. To contrast support and intervention functions, a multilevel linear model was fit using the function type as a fixed effect and allowing for random intercepts per system. Similarly, a 1-way analysis of variance checked for differences in technological sophistication among the four different support types.

#### Developments Over Time

Changes over time could take place both across and within systems. A total of two linear regression models examined development in size and technological sophistication across systems. Moreover, three multilevel linear models allowed studying development within systems. They determined whether size, technological sophistication, and evaluation quality changed across versions. Random intercepts modeled the nested relationship of versions within systems.

## Results

### Characterization

In total, 133 systems with 259 versions were identified. Coding these systems on their key attributes led to the characterization presented in Table 2.

#### Versions

Systems had 2 versions on average, but more than two-thirds (69.2%, 92/133) only had 1 version. Thus, most systems seem to have been developed for a single research project. Only 10 systems had 5 or more versions, for example, The Sadness Program with 13 versions, MoodGYM with 15 versions, and the Well-being Course with 18 versions.

**Table 2:** The distributions over technology-related key attributes of depression support system versions.

Technology			Value
Number of versions^a^ (N=133), mean (SD)			2.0 (2.5)
**Technology (N=259), n (%)**			
	Offline	69 (26.6)	
	World Wide Web	196 (75.7)	
	Email	112 (43.2)	
	Telephone	53 (20.5)	
	Computer	28 (10.8)	
	Text message	17 (6.6)	
	Mobile	16 (6.2)	
	App	14 (5.4)	
	Sensors	7 (2.7)	
	Social media	6 (2.3)	
	Virtual agent	5 (1.9)	
	Interactive voice response	5 (1.9)	
	CD/DVD	5 (1.9)	
	Virtual reality	2 (0.8)	
	Undefined	4 (1.5)	
**Support type (N=259), n (%)**			
	Autonomous	123 (47.5)	
	Therapist	63 (24.3)	
	Professional	32 (12.4)	
	Adjunct	24 (9.3)	
	Admin	14 (5.4)	
	Lay person	3 (1.2)	
Number of function (N=259), mean (SD)			8.4 (4.5)
**Function type (N=259), n (%)**			
	Intervention	246 (95.0)	
	Execution	214 (82.6)	
	Social	175 (67.6)	
	Monitoring	103 (39.8)	
	Planning	22 (8.5)	
**Sophistication (N=259), mean (SD)**			1.6 (0.6)
	Intervention	1.5 (0.8)	
	Execution	1.7 (0.9)	
	Social	1.5 (0.9)	
	Monitoring	2.1 (1.1)	
	Planning	1.8 (1.0)	

^a^Conducted on systems instead of versions.

#### Information and Communication Technology Platforms

The World Wide Web was the most frequently employed platform, with 75.7% (196/259) of the systems providing functionality on the Web and 6.2% (16/259) of the systems providing responsive website content that could also be displayed appropriately on mobile phones. Emails were sent or received in 43.2% (112/259) of systems. Following email, telephone (20.5%, 53/259) and text messages (6.6%, 17/259) were frequently used to reach out to users. Only 1.9% (5/259) of the systems made use of storage media, such as CD and DVD and just as few exhibited technologies such as virtual agents (1.9%, 5/259), virtual reality (0.8%, 2/259), or connected to social media services (2.3%, 6/259).

#### Guidance

E-health software can include various types of human guidance or be entirely autonomous. Approximately half of all systems classified as the latter (47.5%, 123/259). In the remaining systems, guidance was mostly provided by the health care professionals. These were therapists in 24.3% (63/259) of cases and practitioners of related professions, such as coaches, nurses, social workers, or clinical psychology students in 12.4% (32/259) of cases. Less than 10% (24/259) of guided systems were offered as adjunct systems, that is, systems that support face-to-face therapy. A total of 5.4% (14/259) of systems were supported by technicians and other administrators, and only 1.2% (3/259) of systems asked for support by a layperson, typically a peer, friend, or family member of the user.

#### Size and Functionality

In terms of size, the average system offered 8 functions (Mdn=8), with a range from 1 to 21. Furthermore, systems had, on average, 6 modules (Mdn=6) and an intended usage duration of slightly less than 9 weeks (Mdn=8). Although nearly all software contained some intervention functions (95.0%, 246/259) and some support functions (91.5%, 237/259), the four support function types were not equally represented. A total of 82.6% (214/259) of systems included execution support, such as reminders via text message. Social support functionality was provided by 67.6% (175/259) of systems. This was either *direct*, whereby the user communicated with a human, or *indirect*, whereby the user could, for example, see that other people had performed the program before them. The least represented support function type (8.5%, 22/259) was planning support. A typical example of a planning support function was setting up a treatment schedule at the outset of the intervention. Within systems, intervention functions were dominant: systems contained, on average, twice as many intervention functions as support functions (*V*=15,079, *P*<.001, *r*=0.09). In addition, unguided and guided systems differed in their composition, with the former only having 63% of the number of functions of the latter (*F*_1,233_=51.34, *P*<.001, *R^2^*=0.18). This effect showed for both intervention (*U*=3467, *P*<.001, *r*=0.41) and support (*U*=3839.5, *P*<.001, *r*=0.28) functions.

#### Therapeutic Aspects

Although the literature search and filtering focused on systems aiming to reduce depressive symptoms, only 69.9% (181/259) of the identified software targeted depression exclusively. A total of 9.3% (24/259) of these specifically targeted users with a comorbid physical illness (eg, cancer, multiple sclerosis, and diabetes). A few systems supported comorbidities in general (nonspecific, 2.7%, 7/259). Of all systems, 16.6% (43/259) also considered anxiety. However, other mental comorbidities were excluded from the reviewed literature, as they typically formed the primary treatment objective (eg, in systems targeting psychotic conditions and depression simultaneously).

**Figure 3:**
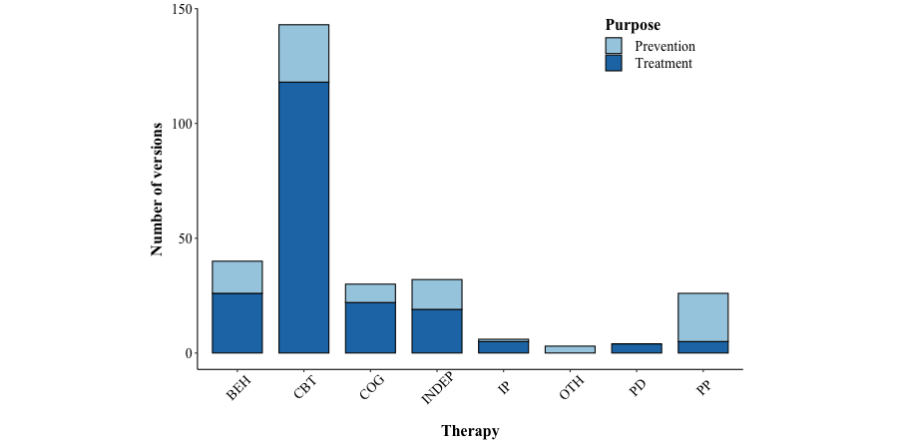
The number of versions with the purpose of preventing or treating depression per therapy. A detailed list of the therapy subtypes for each of the therapy categories listed here can be found in Multimedia Appendix 5. BEH: behavioral therapy, CBT: cognitive behavioral therapy, COG: cognitive therapy, INDEP: independent of specific therapeutic theory, IP: interpersonal therapy, OTH: other, PD: psychodynamic therapy, PP: positive psychology.

The most prominently represented intervention functions, present in 78.9% (194/259) of systems, were unrelated to specific therapies, that is, they could be categorized with many or all different therapies, such as *learning to recognize one’s own symptoms* or *preventing relapses*. A large percentage of software made use of behavioral (62.6%, 154/259), cognitive (58.9%, 145/259), and cognitive behavioral (50.4%, 124/259) functions. Taken together, techniques related to cognitive behavioral therapy (CBT) were present in 84.9% (209/259) systems. Techniques from psychodynamic approaches were rare (2.0%, 5/259), as were life reviewing or hypnosis techniques (together present in 2.8%, 7/259, denoted by *others* in Table 3). A total of 69.5% (180/259) of systems had the purpose of treating depression and 29.3% (76/259) of systems had the purpose of preventing it. Only 1.2% (3/259) of the systems aimed to support patients in maintaining a depression-free state. The system purpose was related to the therapeutic approach (*χ^2^*_7_=34.1, *P*<.001, Nagelkerke *R^2^*=0.24). Systems with Positive Psychology techniques were more often intended for prevention than for treatment. This was not the case for systems with techniques from other therapies (Figure 3).

Similarly, guided systems (χ*^2^*_1_=10.0, *P*=.002, Nagelkerke *R^2^*=0.05) were more often used in treatment systems (n_guided_=105, n_unguided_=75), whereas unguided ones were used more in prevention (n_guided_=28, n_unguided_=48).

**Table 3:** The distributions over therapy-related key attributes of depression support system versions.

Therapy			Value
**Comorbidity (N=259), n (%)**			
	None	181 (69.9)	
	Anxiety	43 (16.6)	
	Physical	24 (9.3)	
	Nonspecific	7 (2.7)	
	Addiction^a^	5 (1.9)	
	Insomia^b^	2 (0.8)	
**Purpose (N=259), n (%)**			
	Treat	180 (69.5)	
	Prevent	76 (29.3)	
	After-care	3 (1.2)	
Duration (weeks; N=210), mean (SD)			8.7 (9.1)
Number of modules (N=218), mean (SD)			5.9 (3.5)
**Therapy class (N=259), n (%)**			
	Independent	194 (78.9)	
	Behavioral	154 (62.6)	
	Cognitive	145 (58.9)	
	Cognitive behavioral therapy	124 (50.4)	
	Interpersonal	43 (17.5)	
	Positive psychology	43 (17.5)	
	Psychodynamic	7 (2.0)	
	Other	5 (2.8)	

^a^Addiction is separated from physical illness, as it can be regarded as both a physical and a mental illness.

^b^Insomnia is separated from physical illness, as insomnia is also a symptom of depression.

#### Evaluation

Systems were often evaluated only once with end users (86.9%, 225/259) and, for the largest part, in comparative trials (77.2%, 200/259). In controlled trials, attention control (41.7%, 73/175) and waitlist (39.4%, 69/175) were similarly common, whereas treatment as usual (28.6%, 50/175) was less frequent (Table 4). In total, 72.2% (187/259) of systems were evaluated in controlled trials. Multimedia Appendix 7 comprises two tables ranking systems according to the number of evaluations and the total number of participants who participated in these studies.

Although 21 different measures assessed depressive symptomatology across studies, the most frequent by far were the Patient Health Questionnaire [26], Beck’s Depression Inventory [27], and the Center for Epidemiological Studies Depression Scale [28]. An additional 11 measures were depression related, determining such things as fatigue, rumination, stress, or quality of life. Finally, 12.0% (31/259) of systems were evaluated in studies having primary outcomes other than depression, such as usability.

**Table 4:** The distributions over evaluation-related key attributes of depression support system versions.

Evaluation		Value
Number of studies, mean (SD)		1.2 (0.9)
**Quality (N=259), n (%)**		
	Comparative	200 (77.2)
	Noncomparative	74 (28.6)
**Control group types (N=259), n (%)**		
	Attention controlled	73 (41.7)
	Waitlist	69 (39.4)
	TAU^a^	50 (28.6)
**Measures (N=259), n (%)**		
	PHQ^b^	90 (34.7)
	BDI^c^	74 (28.6)
	CES-D^d^	65 (22.0)
	Other depression measure	57 (25.1)
	Nondepression measure	31 (12.0)

^a^TAU: treatment as usual.

^b^PHQ: Patient Health Questionnaire.

^c^BDI: Beck Depression Inventory.

^d^CES-D: Center for Epidemiological Studies Depression.

#### Description of a Fictional, Prototypical System

For illustration purposes, we outline here a fictional, prototypical depression treatment system by combining insights from the qualitative reading of the articles and the quantitative analyses. This is intended to serve as a narrative description of the taxonomy provided in Multimedia Appendix 5. However, it must be noted that this is a simplification and much variation exists among the systems. A prototypical system takes a CBT approach and might comprise 6 modules, one of which is released every week. The modules can be accessed on a website. The participant is made aware of the presence of a new module via email; thus, the participant is reminded to adhere to the treatment. Modules might cover topics such as activity scheduling, learning to detect automatic thoughts, cognitive restructuring, problem solving, psychoeducation concerning depression and the therapeutic approach, and relapse prevention. Each module comes with exercises that are submitted to be checked by a therapist or similar, who again provides feedback via email. The website might include a small calendar application for the purposes of activity scheduling and a diary application for the purposes of thought recording. In these applications, the user can enter and save information. Once a week, the participant is asked to complete a depression scale, and the therapist is notified if suicidal ideation is detected. The remaining questions are averaged and presented to the user as a mood graph on the landing page. This sketched system would have an average eHDTS score of around 2. For each of the eHDTS levels, a similar, fictional description of possible functions scoring at this level can be found in Multimedia Appendix 8. This is intended to provide a more concise and tangible description than Multimedia Appendix 6 can and to further concretize the taxonomy presented in Multimedia Appendix 3.

### Technological Sophistication

#### Systems

**Figure 4:**
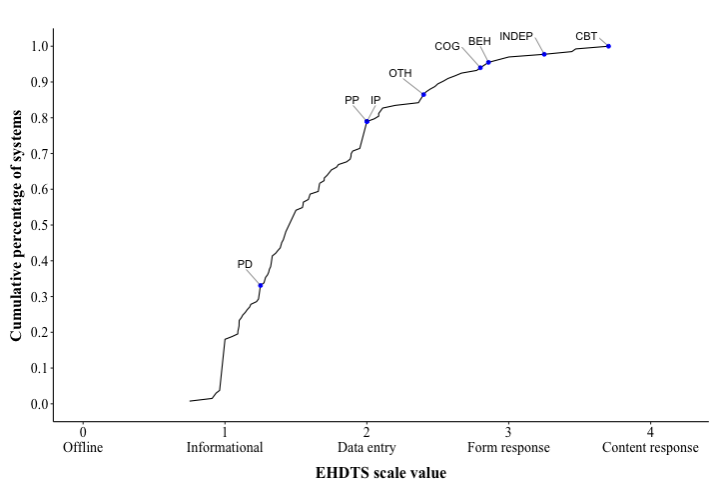
Cumulative density plot of all systems over the e-mental Health Degree of Technological Sophistication (eHDTS) scale. This analysis was conducted on the unweighted average of technological sophistication of the systems. Labeled dots show the highest scoring system within a specific therapy, as indicated by the label. BEH: behavioral therapy, CBT: cognitive behavioral therapy; COG; cognitive therapy; eHDTS: e-mental Health Degree of Technological Sophistication; INDEP: independent of specific therapeutic theory; IP: interpersonal therapy; Oth: other; PD: psychodynamic therapy; PP; positive psychology.

The average system comprised, to a large extent, functions providing information to the user without collecting and interpreting information from the user. This is further detailed in Figure 4. Almost all interventions had the majority of their functions delivered through technology, that is, hardly any system scored below 1 on technological sophistication. However, only 21.1% (28/133) of systems had a sophistication level above 2, indicating that they were responsive to activities and information coming from the user. These systems comprised, for the most part, interventions inspired by CBT or closely related therapies. In fact, CBT systems lead the list of the most technologically advanced systems, even when adjusting for the number of functions (Table 5). The top two systems in both rankings are Help4Mood [29] and Deprexis [30]. The latter is a commercial system aiming to mimic the structure of face-to-face CBT therapy, whereas the former is a self-monitoring system that includes a virtual conversational agent. Both presented high-tech solutions according to the eHDTS scale, as they adapted the intervention to the users’ indicated interests and needs (Deprexis) or to the self-monitoring data from users (Help4Mood). To allow researchers to compare their own system, Multimedia Appendix 9 provides the eHDTS score per cumulative percentage decile of systems for both the weighted and unweighted system means. That is, when knowing the average weighted or unweighted eHDTS score of their system, researchers can use the table to determine which decile of systems their system scores at, below, or above.

Technological sophistication was not linked to the number of functions (*r*_257_=0.01, *P*=.83), the system purpose (*χ^2^*_1_=0.2, *P*=.69), or guidance (*χ^2^*_1_=3.0, *P*=.08). However, it did relate to the evaluation quality (*χ^2^*_1_=6.1, *P*=.01, Nagelkerke *R^2^*=0.03). More technologically sophisticated systems were less likely (OR 0.59) to have been evaluated in comparative trials than less technologically sophisticated systems. Furthermore, when regarding specifically randomized controlled trials (RCTs), we found that 80.8% (139/172) of RCTs evaluate systems that score below data entry level on average, with the respective percentage of RCTs per eHDTS interval being the following: [0,1)—4%; [1,2)—77%; [2,3)—16%; and [3,4)—3%.

**Table 5:** Ranking of the 10 systems with the highest degree of technological sophistication in the database, first based on average e-mental Health Degree of Technological Sophistication (eHDTS) score (M) and then based on a weighted eHDTS score (M_w_), trading off eHDTS against the number of functions in a system. The analyses were conducted on the basis of systems rather than versions. We advise some caution in taking this table at face value, as it is based on the aggregated eHDTS scores with some of the scales only having moderate interrater agreement.

Rank	Unweighted					Weighted				
	System	Therapy	*n*_f_^a^	*M* ^b^	System		Therapy	*n*_f_	*M*	*M*_w_^c,d^
1	Help4Mood [29]	CBT^e^	13.5	3.70	Help4Mood [29]		CBT	13.5	3.70	2.31
2	Deprexis [30]	CBT	14.3	3.47	Deprexis [30]		CBT	14.3	3.47	2.31
3	MOSS App [31]	CBT	9	3.44	Buhrman [32]		CBT	20	1.95	1.85
4	Ahmedani [33]	MI^f^, CBT	4	3.25	Building a Meaningful Life through BA^g^ [34]		BA	20	1.90	1.81
5	DCAT ATA [35]	SM^h^	5	3.00	Shamekhi [36]		MFN^i^	13	3.00	1.80
6	Shamekhi [36]	MFN	13	3.00	Living to the full [37]		ACT^j^	14.5	2.62	1.77
7	Panoply [38]	CBT	7	2.86	MindBalance [39]		CBT	14	2.57	1.67
8	MyPAA [40]	PhA^k^	7	2.86	Space from Depression [41]		CBT	15	2.20	1.54
9	EVO [42]	CCT^l^	5	2.80	Mobilyze! [43]		BA	13	2.54	1.52
10	Daybuilder [44]	SM	6.5	2.77	MOSS App [31]		CBT	9	3.44	1.38

^a^n_f_: number of functions.

^b^M: unweighted average.

^c^M_w_: weighted average.

^d^To obtain the weighted average (M_w_), the unweighted average (M) is weighted with the feature scaled number of functions (nf): Mw=M(nf – min(nf))/(max(nf)-min(nf)), with min(nf)=1 and max(nf)=21.

^e^CBT: cognitive behavioral therapy.

^f^MI: motivational interviewing.

^g^BA: behavioral activation.

^h^SM: symptom monitoring.

^i^MFN: mindfulness.

^j^ACT: acceptance and commitment therapy

^k^PhA: physical activity.

^l^CCT: cognitive control training.

#### Functions

Support functions (mean 1.73, SD 1.06) scored higher in technological sophistication than intervention functions (mean 1.43, SD 0.88), although this effect was small (*F*_1,1903_=38.11*, P*<.001, Level1 *R^2^*=0.03). An equally small effect was observed while comparing the 4 types of support functions on their technological sophistication (*F*_3,619_=8.46, *P*<.001, Level1 *R^2^*=0.04). Monitoring support functions had the highest average degree of technological sophistication (Table 2). This indicates that monitoring functions were mostly technologically sophisticated to the extent that they reported data back to the user, but they neither interpreted data nor used the data to adapt the intervention. Social support and intervention functions ranked the lowest in terms of technological sophistication (Table 2). In social support, the score translates to technology being typically either used to simply provide contact information to the user or to serve as a communication medium between human support and user. Intervention functions often took an informational form, possibly with a limited amount of interactivity, for example, clicking through pages or filling in a Web-based diary.

**Figure 5:**
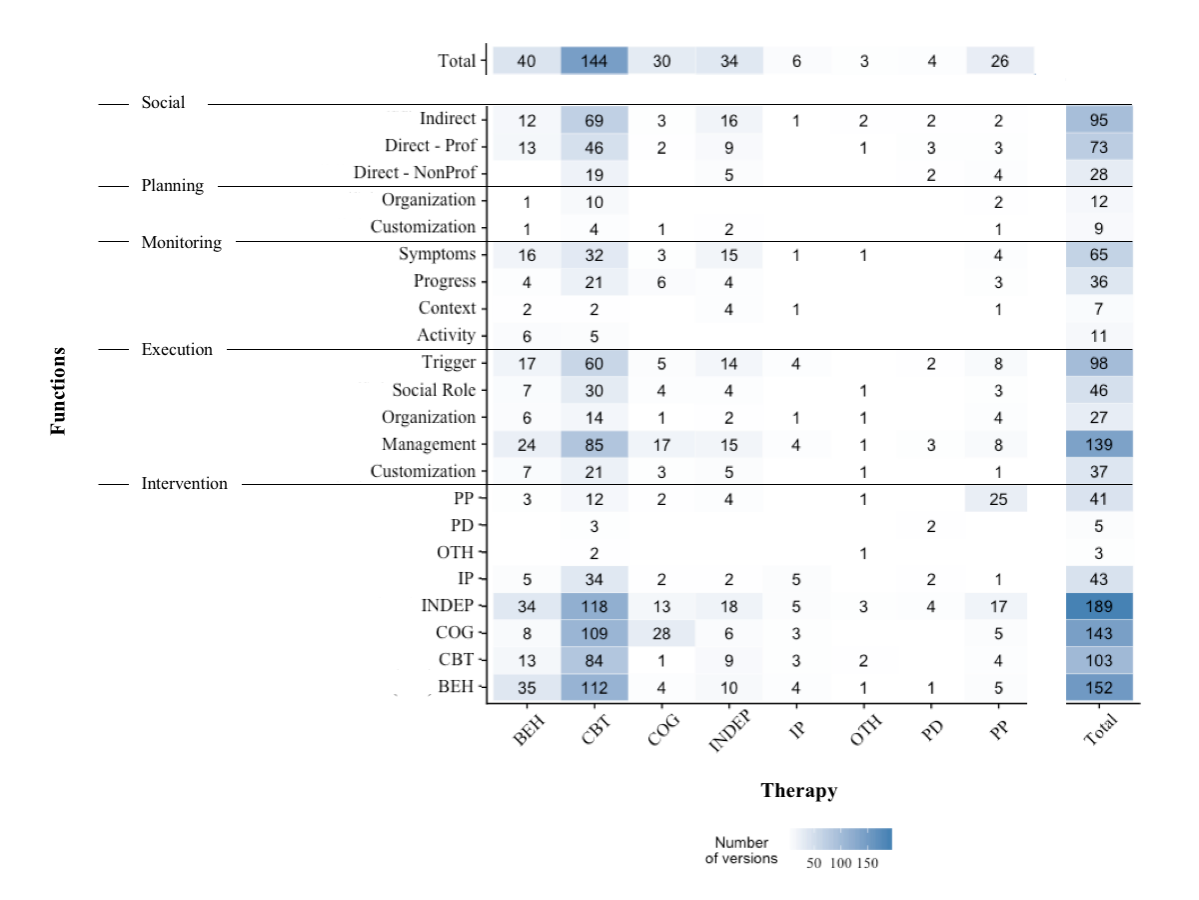
Heatmap of the frequency with which a specific type of function was implemented in a therapy across all systems of that therapy. BEH: behavioral therapy, CBT: cognitive behavioral therapy, COG: cognitive therapy, INDEP: independent of specific therapeutic theory, IP: interpersonal therapy, OTH: other, PD: psychodynamic therapy, PP: positive psychology.

**Figure 6:**
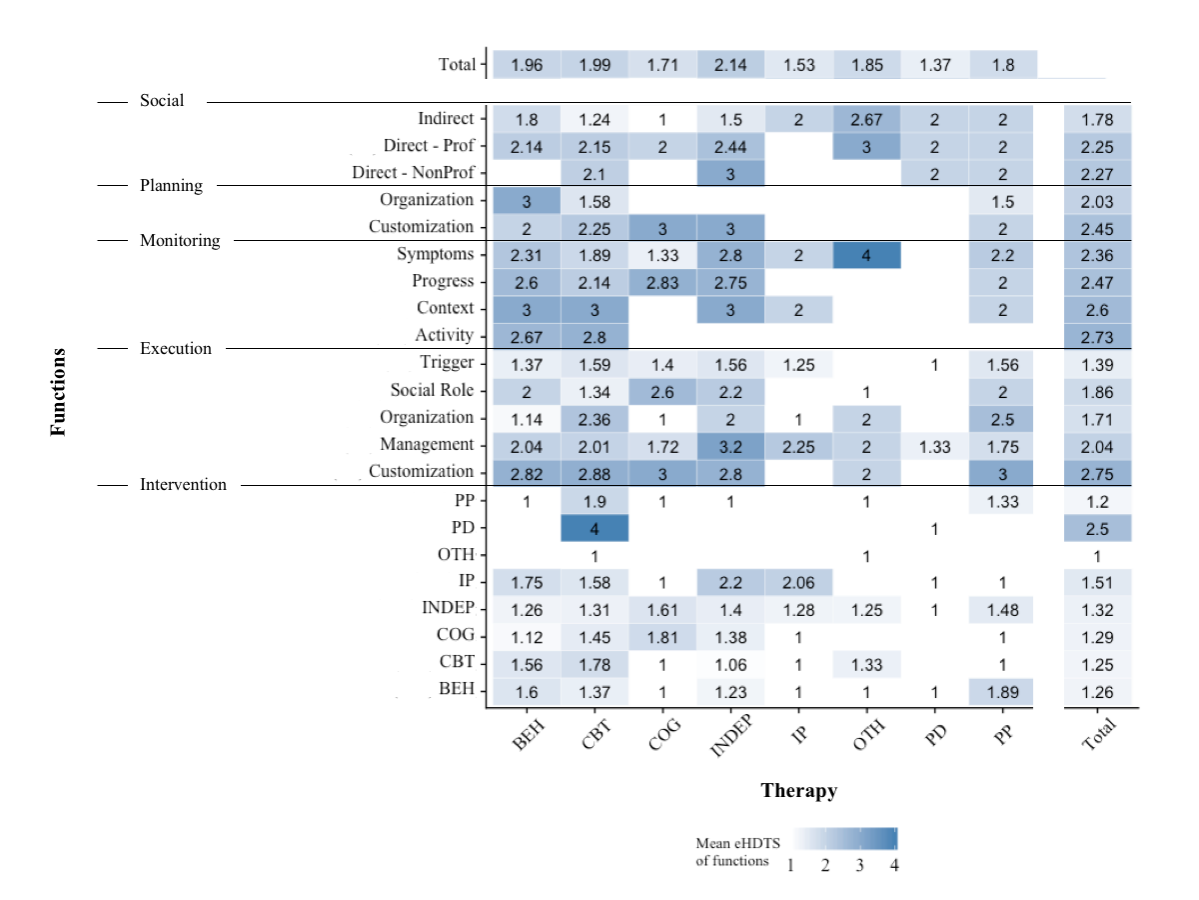
Heatmap of the average degree of technological sophistication per function type and therapy. BEH: behavioral therapy, CBT: cognitive behavioral therapy, COG: cognitive therapy, INDEP: independent of specific therapeutic theory, IP: interpersonal therapy, OTH: other, PD: psychodynamic therapy, PP: positive psychology.

The most frequently implemented support functions were execution support pertaining to the management of user progress and risk, triggers, indirect social support, professional direct social support, and symptom monitoring (Figure 5). However, only management execution support and indirect social support were present at least once in systems of all different therapies. A barely implemented function type was planning support. Shifting the focus to intervention functions, most stem from CBT or related therapies or are independent of a specific therapeutic framework. CBT systems clearly dominate the field, with most of the different function types being implemented in numerous such systems (Figure 5). Yet, the average technological sophistication of functions (Figure 6) was not related to how frequently they were implemented (*r*_154_=0.12, *P*=.12). Thus, functions that are often implemented are neither more nor less technologically sophisticated, on average, than functions that are rarely implemented. However, the more often a function was implemented, the more often at least 1 of these implementations was responsive to interaction activity of the user, for example, time spent on platform, or even to the content of information provided by the user (*r*_154_=0.43, *P*<.001). For interested readers, Multimedia Appendix 10 finally also demonstrates that nearly all of the different functions were implemented in a highly sophisticated manner in at least one system.

### Developments Over Time

**Figure 7:**
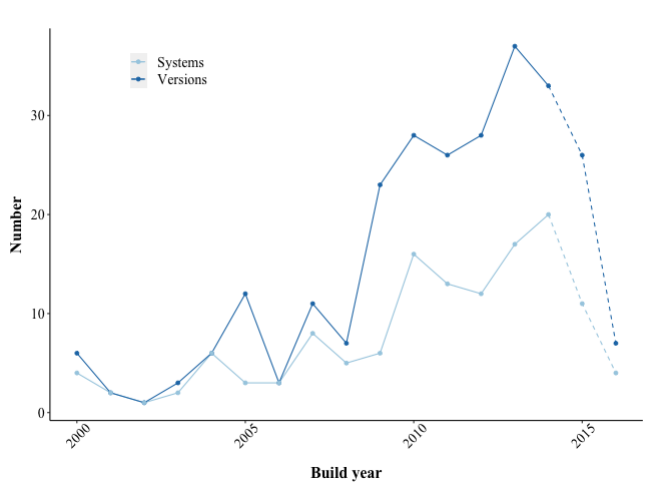
The number of systems and versions developed per year between 2000 and 2016.

**Figure 8:**
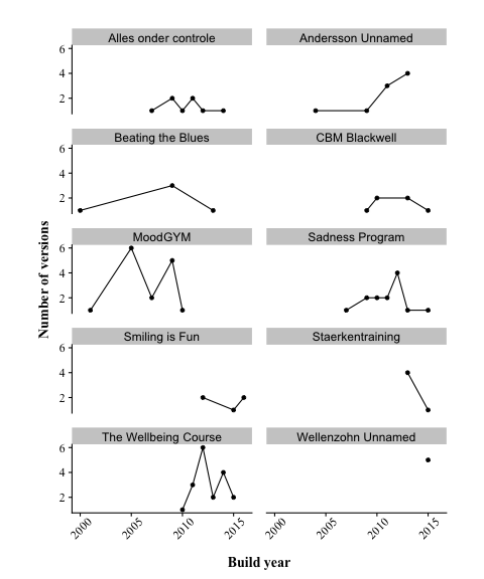
The number of versions developed per year between 2000 and 2016 for the ten systems having five or more versions.

In the past 2 decades, the field of e-mental health for depression has seen marked growth, with 5 times as many systems developed in 2014 as in 2000 (Figure 7). As several years typically lie between development and the publication of study outcomes, less emphasis may be given to numbers after 2014. The figure also demonstrates that systems were being reused and extended to a substantial degree only from approximately 2009 onward. This is further supported, when examining systems with at least five versions more closely (Figure 8). Only MoodGYM had evolved multiple versions before 2009. Different versions developed within the same year are an indication that they were created for the same study, often differing in only 1 function as an experimental manipulation.

Despite growth in the field in general, systems seemed to neither get larger (*F*_1,257_=0.25, *P*=.62) nor more sophisticated (*F*_1,257_=1.88, *P*=.17) with time. Within systems, growth was observed across versions, with each new version of a system having half of a function more than the previous one (*b*=0.50, *F*_1,125_=11.60, *P*<.001, Level1 *R^2^*=0.06). However, technological sophistication seemed to remain the same (*F*_1,125_=1.96, *P*=.16). Finally, the evaluation quality showed no relationship with the version number (*F*_1,136_=0.07, *P*=.79). Later versions therefore appeared to be no more or less frequently evaluated in comparative trials than earlier ones.

## Discussion

### Principal Findings

Some limitations should be kept in mind when considering implications of the results. The first pertains to the coding of technological sophistication. Namely, the one-dimensional nature of the eHDTS scales can limit them in covering the full extent of the degree to which they reflect how technologized a function is. In monitoring support functions, for example, the scale captures how the system deals with the collected information but not how the data are obtained in the first place. Thus, whether monitoring data are collected via self-report or sensing does not influence the level of technological sophistication. However, as sensors and data analysis methods are becoming increasingly reliable, sensing will likely begin to play a crucial role in more automated, that is, more technologically advanced, systems [45]. In addition to monitoring, this is particularly to be expected in diagnosis and assessment systems [46,47], which we have excluded in this review. Thus, in the future and especially when wishing to also study such systems, the manner in which data are collected should receive more attention in the monitoring scale. An additional point to consider when interpreting the results is the moderate reliability of some high-inference attributes. Although this is a limitation that might influence more detailed findings, such as the exact ranking of the systems according to their eHDTS score, we do not expect it to substantially affect the larger patterns found. However, by double coding samples, we have insight into the reliability of the estimates. In selecting a sample size for double coding, we have aimed for a 10% margin of error for the reliability estimates, as suggested in [48] (see [11] for 95% CI information for each estimate). Two final limitations concern the scope of the reviewed systems, as well as the scope of the reviewed functions. Systems developed for children and adolescents, women with depression during or following pregnancy, and those with comorbid psychiatric conditions, as well as systems developed before 2000, were excluded. In addition, we did not consider commercial systems that are not reported in the scientific literature. How well our findings generalize to these types of systems is therefore open to further investigation. As far as the scope of the reviewed functions is concerned, functions pertaining to data security or the integration of the system with existing health software were not covered. Such aspects of the interventions were typically not found to be reported in the publications. As data security is becoming an important concern of software development and usage, we see a need for more consistent system reporting guidelines and an opportunity for reviews of future systems to subsequently investigate such functionality. In spite of these limitations, the outcomes of the analyses highlight some of the challenges and opportunities for the field of e-mental health for depression.

First, no clear progress in terms of system sophistication was observed between 2000 and 2017, within or across systems. A possible challenge for progress might lie in the short-term approach to system development in the field. In a long-term approach, multiple versions with substantial changes in functionality could be expected. Early versions would be tried in pilot studies, improved, and only eventually tested in an RCT. However, this is not what we found. Despite often proving effective in RCTs, two-thirds of the systems are not evolved and retested (eg, [49-51]). In addition, in systems that do have multiple versions, systems are often extended only by a function for hypothesis testing among versions, and versions do not differ in technological sophistication. Finally, there was also no association between the evaluation level and the version number for systems that had more than 1 version.

Another challenge for the field is posed by the spread in technological sophistication. Our analyses confirmed what has been hinted at in previous reviews and meta-analyses [2,6,7]: systems developed within a research context vary in their implementation and in their technological sophistication. By and large, they are not very technologically advanced, and those systems that are mostly informational in nature account for 81% of what is evaluated in RCTs. Only approximately one-fifth of the systems have a substantial amount of functions that are responsive to input from the user. These differences in technological realization have, thus far, been neglected in literature syntheses taking a clinical psychology perspective. For example, two effects identified in such syntheses are that both adherence and effect size appear to increase with higher levels of human guidance (no guidance vs administrative guidance vs therapist guidance) [3]. Although this has been hypothesized to be linked to missing therapeutic alliance or accountability, our results indicate another possibility. We found the lack of guidance to be neither compensated with more content or technological support nor with a more responsive and, thus potentially more engaging, system. It is therefore possible that guidance plays a role, especially when systems are not very responsive. As, according to our analyses, this applies to approximately 80% of the systems, the results of meta-analyses over all systems may not generalize to more technologically advanced solutions. This notion finds some support in a system-specific meta-analysis of the Deprexis system [52], which ranked second in our ranking of the most technologically advanced systems. Across different studies with Deprexis, dropout ranged from 6% to 50%, contrasting with the average dropout rate of 74% found for other unguided systems in general [3]. Furthermore, it was not only observed that unguided Deprexis had an average effect size across trials comparable with that of other systems, including administrative guidance [3], but also that adding guidance did not influence the magnitude of the effect. However, it must also be emphasized at this point that the potential of more technologically advanced systems leading to higher adherence is merely a hypothesis that is in need of further investigation.

Aside from these challenges, we also see opportunities. Systems developed for depression, to date, are hardly making use of the full bandwidth of available technology. In fact, empirically evaluated systems are mostly delivered on the World Wide Web. Only a very few take a mobile form as either native apps or cross-platform Web applications. This is surprising considering that smartphones became a ubiquitous and highly used technology approximately mid-way of the examined time period. In a review from 2015 on the state of the app marketplace for depression apps, 82 apps had been identified for the treatment of depression [53]. A later review (2017) found that only 5 apps for depression treatment had been empirically evaluated in effectiveness trials [54]. Therefore, an abundance of apps exists, but most apps are commercial, and few have been scientifically studied. However, the empirically evaluated apps included in this review fared well in technological sophistication, such as Mobilyze! [43] and Mobile Sensing and Support (MOSS) [31]. Both apps attempt to learn how to provide context-sensitive interventions on the basis of phone sensor readings. The former uses models trained before delivering the interventions, whereas the latter continuously learns user preferences as it intervenes. In addition to mobile apps, there are several other underexplored innovative technologies, such as social media, conversational agents, and virtual reality. Yet, where these were implemented, some technologically interesting solutions emerged. In social media systems, Panoply [38] can be considered a technological forerunner. It integrates social networking between Panoply users and crowdsourcing from Amazon Mechanical Turk to ensure high-quality content, both in terms of users’ thought-recording posts and in terms of responses to these posts. Woebot [55], a fully autonomous chatbot provided on social media, was developed after our search; therefore, it was not included in the analyses. Through short, daily conversations using Facebook instant messenger, Woebot continuously checks in with users and tailors short intervening information and empathic replies to their reported mood. Finally, a creative attempt to alleviate depression is presented by the only virtual reality system that we found [56]. Users are first asked to comfort a virtual avatar with the embodiment of a child. They then take on the perspective of this child in virtual reality to hear their own comforting words said back to them, with the effect of increasing their self-compassion. However, innovative technology solutions, such as the ones mentioned, are scarce. Thus, there still are many opportunities for the field to explore such directions.

### Conclusions

The e-mental health field, focusing specifically on the treatment and prevention of depressive disorders, is large and consist of a very active research community, as evidenced by the vast body of literature that could be identified for this study. In line with our research questions, three main conclusions can be drawn. First, although the system landscape is overall varied, there are clear trends: three quarters of the systems implement therapeutic techniques related to CBT, three quarters are delivered on the World Wide Web, and three quarters have been evaluated in comparative trials. Second, most systems do not get close to the full technological potential of e-mental health. However, some do get close. On the level of functions, we have further found that nearly all functions have been implemented in a responsive manner in at least one system, showing that the high end of the scale is obtainable across the board. Third, there appears to be no clear technological development across systems between 2000 and 2017. Furthermore, within systems that have multiple versions, a small increase in size with each new version showed, but it was not the case in technological sophistication. Consequently, it can be argued that, from a technological perspective, there is still room for improvement. Future research investigating the relationship between software implementation and clinical outcomes will need to show whether such improvement is beneficial and cost-efficient with regard to development and maintenance.

To conclude, the scientific contribution of this research is its provision of a comprehensive overview of the technological state of the art of e-mental health systems for the prevention and treatment of adult major depressive disorder, developed and studied since the year 2000. This is further accompanied by EHealth4MDD, an open-access database containing all extracted and coded information from the literature used in this writing. Together, the review and database are intended to serve as inspiration for the development of new systems on the one hand and as facilitators for the study of hypotheses related to system composition, on the other hand.

## Appendix

Multimedia Appendix 1 The search terms that were used in retrieving primary articles from the databases Scopus, PubMed, and Web of Science are shown. Columns are combined with logical ANDs, whereas cells within columns are combined with logical ORs. The first 2 columns were searched for within titles and keywords; the third and fourth column were searched for within abstracts. Terms from the Exclude column were not allowed to appear within titles only. The first and second column include in italics the Medical Subject Heading terms that were used in addition to the regular search terms in PubMed. Finally, where possible, wildcards were used to expand the search terms with * denoting any string, including the empty one, and $ denoting any string of length 1 or the empty string.

Multimedia Appendix 2 List of all publications that were included in this review.

Multimedia Appendix 3 Taxonomy of software systems for depression. This was inspired by the one for conversational agents, presented in Montenegro JL, da Costa CA, and da Rosa Righi R. “Survey of conversational agents in health.” Expert Systems with Applications (2019). It is important to note that this is not an exact graphical representation of all concepts in the database but is intended as an illustration of the most descriptive attributes of software systems for depression. For readers interested in an exact graphical representation of the database, we refer to the SQL schema diagram on the EHealth4MDD (database) website.

Multimedia Appendix 4 Categorization of support functions at level 0 (L0), level 1 (L1), and level 2 (L2). Customization as an Execution function type means that the system or intervention could be altered throughout the usage period according to the user’s preferences, while as a Planning function type, customization was only possible at the start of the intervention. The difference between Management and Organization within the Execution type is that Organization pertains to management of aspects of the specific system and intervention, whereas Management pertains to dealing with higher-level problems or aspects.

Multimedia Appendix 5 The categorization of therapeutic frameworks into therapies (L1). An example function at level 0 (L0) is provided for each therapeutic framework. Some of the therapies were mentioned by authors as having influenced the design of the system, but using our classification, no intervention functions pertaining to the therapy could be found. Therefore, no example can be given. This does not mean that no functionality reflecting the therapy was implemented, for example, a symptom monitoring approach might well result in functionality to aid in the monitoring of symptoms. However, with our classification, this would be classified as a monitoring support function rather than as an intervention function. Similarly, influences from Social Cognitive Theory may have found their way into the system in the form of vignettes, which we classify as indirect social support functions rather than intervention functions.

Multimedia Appendix 6 Part of the EHealth4MDD (database) website, detailing the 5 different subscales of e-mental Health Degree of Technological Sophistication. Exact level definitions for each of the scales and an example function are provided.

Multimedia Appendix 7 A total of 2 tables showing the ranking of the 133 systems contained in the database by evidence base, as far as this has been recorded in the database. The first table shows the evidence base of systems evaluated in comparative trials (such that are randomized, controlled, or both), whereas the second table shows the evidence base of systems evaluated in noncomparative trials (single-group trials). Both tables are sorted first on the number of evaluations, on the number of participants recruited to take part in the study (sum over all study arms), and the number of participants who completed the study (not including follow-up). For readers interested in more information, the system key, as denoting systems in the database, is provided. This should allow for easy querying of associated versions, authors, and articles, to name only a few things.

Multimedia Appendix 8 Table providing a description of some possible functions that a system at each of the e-mental Health Degree of Technological Sophistication (eHDTS) score levels might have. These descriptions are fictional, and eHDTS scores at the system level are averages.

Multimedia Appendix 9 Deciles and their corresponding scale values for the weighted and unweighted scale, for example, 50% of systems have an average technological sophistication of 1.5 or lower. The weighted column takes into account the number of functions that a system implements.

Multimedia Appendix 10 Heatmap of the maximum degree of technological sophistication per function type and therapy. This gives insight into the technological state of the art of each function type and therapy.

## Abbreviations

CBT: cognitive behavioral therapy
eHDTS: e-mental Health Degree of Technological Sophistication
ICT: information and communication technology
RCT: randomized controlled trial
