# Supplementary material for: Technological State of the Art of Electronic Mental Health Interventions for Major Depressive Disorder: Systematic Literature Review
Source: J Med Internet Res. 2020 Jan 20;22(1):e12599. doi: 10.2196/12599 (PMC6997926; doi:10.2196/12599)
Supplement: Multimedia Appendix 1 [file jmir_v22i1e12599_app1.pdf]

Technology	Condition	Goal	Evaluation	Exclude
computer*/*Computers*/*Computer Systems*	depress*/*depressive disorder*/*depressive disorder, major*	prevent*	evaluat*	PTSD
internet/*Internet*	dysthmi*/*dysthymic disorder*	treat*	trial	postpartum
Software		train*	test	postnatal
online		monitor*	study	perinatal*
web*/*Web Browser*		simulat*	pilot	adolescent*
mail*		interven*	rct	child*
mobile/*Cell Phones*/*Computers, Handheld*		support*	conduct*	telephone
phone			experiment*	tele$health
text message			significan*	review
app			allocat*	meta$analy*
techn*			control*	survey
digital			participant*	screening
automat*			random*	psychosis
virtual			recruit*	psychotic
remote				bipolar
self$help/*Self-Help Devices*				
cCBT/*Therapy, Computer-Assisted*				
iCBT				

PubMed

(((internet[Title] OR online[Title] OR web*[Title] OR mobile[Title] OR computer*[Title] OR techn*[Title] OR telemedic*[Title] OR tele-medic*[Title] OR virtual[Title] OR remote[Title] OR e-health[Title] OR ehealth[Title] OR automat*[Title] OR "text message"[Title] OR mail*[Title] OR mhealth[Title] OR m-health[Title] OR digital[Title] OR phone[Title] OR app[Title] OR cCBT[Title] OR distance[Title] OR self-help[Title]) OR (Internet[MeSH Term] OR "Web Browser"[MeSH Term] OR "Therapy, Computer-Assisted"[MeSH Term] OR Software[MeSH Term] OR "Computers, Handheld"[MeSH Term] OR Telemedicine[MeSH Term] OR "Cell Phones"[MeSH Term] OR "Self-Help Devices"[MeSH Term] OR "Computer Systems"[MeSH Term] OR Computers[MeSH Term]) OR (internet[Other Term] OR online[Other Term] OR web*[Other Term] OR mobile[Other Term] OR computer*[Other Term] OR techn*[Other Term] OR telemedic*[Other Term] OR tele-medic*[Other Term] OR virtual[Other Term] OR remote[Other Term] OR ehealth[Other Term] OR e-health[Other Term] OR automat*[Other Term] OR "text message"[Other Term] OR mail*[Other Term] OR mhealth[Other Term] OR m-health[Other Term] OR digital[Other Term] OR phone[Other Term] OR app[Other Term] OR cCBT[Other Term] OR distance[Other Term] OR self-help[Other Term])) AND ((depress*[Title] OR dysthymi*[Title]) OR (depressive disorder[MeSH Terms] OR depressive disorder, major[MeSH Terms] OR dysthymic disorder[MeSH Terms]) OR (depress*[Other Term] OR dysthymi*[Other Term])) AND ((prevent*[Title/Abstract] OR treat*[Title/Abstract] OR monitor*[Title/Abstract] OR train*[Title/Abstract] OR simulat*[Title/Abstract] OR interven*[Title/Abstract] OR support*[Title/Abstract]) AND (evaluat*[Title/Abstract] OR trial[Title/Abstract] OR test[Title/Abstract] OR pilot[Title/Abstract] OR study[Title/Abstract] OR RCT[Title/Abstract] OR conduct*[Title/Abstract] OR experiment*[Title/Abstract] OR significan*[Title/Abstract] OR allocat*[Title/Abstract] OR control*[Title/Abstract] OR random*[Title/Abstract] OR recruit*[Title/Abstract] OR participant*[Title/Abstract])) NOT (PTSD[Title] OR postpartum[Title] OR postnatal[Title] OR adolescent*[Title] OR child*[Title] OR bipolar[Title]) AND ((Classical Article[ptyp] OR Clinical

Conference[ptyp] OR Clinical Study[ptyp] OR Clinical Trial[ptyp] OR Comparative Study[ptyp] OR Controlled Clinical Trial[ptyp] OR Evaluation Studies[ptyp] OR Journal Article[ptyp] OR Observational Study[ptyp] OR Pragmatic Clinical Trial[ptyp] OR Randomized Controlled Trial[ptyp] OR Technical Report[ptyp] OR Validation Studies[ptyp]) AND hasabstract[text] AND ("2000/01/01"[PDat] : "2017/12/31"[PDat])))

Scopus
((((((TITLE (internet OR online OR web* OR mobile OR computer* OR techn* OR virtual OR remote OR e?health OR "text message" OR mail* OR m?health OR digital OR phone OR app OR ccbt OR icbt) OR KEY (internet OR online OR web* OR mobile OR computer* OR techn* OR virtual OR remote OR e?health OR "text message" OR mail* OR m?health OR digital OR phone OR app OR ccbt OR icbt)) AND (TITLE (depress* OR dysthymi*) OR KEY (depress* OR dysthymi*))) AND ABS (prevent* OR treat* OR monitor* OR train* OR simulat* OR interven* OR support*)) AND ABS (evaluat* OR trial OR test OR pilot OR study OR rct OR conduct* OR experiment* OR significan* OR allocat* OR control* OR random* OR recruit* OR participant*)) AND (PUBYEAR > 2000)) AND NOT (TITLE (ptsd OR postpartum OR postnatal OR perinatal OR adolescent* OR child* OR bipolar OR review OR meta?analy* OR survey OR screen* OR assess* OR psychosis OR telephone OR tele?health OR tele?medicine OR addict* OR predict* OR stigma OR schizophreni* OR reproduct* OR maternal OR sleep OR anti$depress* OR "support group") OR KEY (ptsd OR postpartum OR postnatal OR perinatal OR adolescent* OR child* OR bipolar OR review OR meta?analy* OR survey OR screen* OR assess* OR psychosis OR telephone OR tele?health OR tele?medicine OR addict* OR predict* OR stigma OR schizophreni* OR reproduct* OR maternal OR sleep OR anti$depress* OR "support group")))) AND (LIMIT-TO (DOCTYPE , "ar ") OR LIMIT-TO (DOCTYPE , "cp ") OR LIMIT-TO (DOCTYPE , "ip ")) AND (LIMIT-TO (LANGUAGE , "English "))

Web of Science
TS=(internet OR online OR web* OR mobile OR computer* OR techn* OR virtual OR remote OR e?health OR "text message" OR mail* OR m?health OR digital OR phone OR app OR ccbt OR icbt) AND TS=(depress* OR dysthymi*) AND TS=(prevent* OR treat* OR monitor* OR train* OR simulat* OR interven* OR support*) AND TS=(evaluat* OR trial OR test OR pilot OR study OR rct OR conduct* OR experiment* OR significan* OR allocat* OR control* OR random* OR recruit* OR participant*) NOT (TI=(ptsd OR postpartum OR postnatal OR perinatal OR adolescent* OR child* OR bipolar OR review OR meta?analy* OR survey OR screen* OR assess* OR psychosis OR telephone OR tele?health OR tele?medicine OR addict* OR predict* OR stigma OR schizophreni* OR reproduct* OR maternal OR sleep OR anti$depress* OR "support group") OR TS=(ptsd OR postpartum OR postnatal OR perinatal OR adolescent* OR child* OR bipolar OR review OR meta?analy* OR survey OR screen* OR assess* OR psychosis OR telephone OR tele?health OR tele?medicine OR addict* OR predict* OR stigma OR schizophreni* OR reproduct* OR maternal OR sleep OR anti$depress* OR "support group")) AND LANGUAGE: (English)Refined by: DOCUMENT TYPES: (ARTICLE OR PROCEEDINGS PAPER) AND WEB OF SCIENCE CATEGORIES: (ONCOLOGY OR PSYCHIATRY OR GERONTOLOGY OR PSYCHOLOGY CLINICAL OR COMPUTER SCIENCE INFORMATION SYSTEMS OR PUBLIC ENVIRONMENTAL OCCUPATIONAL HEALTH OR HEALTH CARE SCIENCES SERVICES OR ENGINEERING ELECTRICAL ELECTRONIC OR INTEGRATIVE COMPLEMENTARY MEDICINE OR PSYCHOLOGY MULTIDISCIPLINARY OR COMPUTER SCIENCE THEORY METHODS OR SUBSTANCE ABUSE OR BEHAVIORAL SCIENCES OR MEDICINE GENERAL INTERNAL OR REMOTE SENSING OR PSYCHOLOGY EXPERIMENTAL OR PSYCHOLOGY OR PSYCHOLOGY APPLIED OR NURSING OR MEDICAL INFORMATICS OR SOCIAL SCIENCES

INTERDISCIPLINARY OR MEDICINE RESEARCH EXPERIMENTAL OR COMPUTER SCIENCE ARTIFICIAL INTELLIGENCE OR COMPUTER SCIENCE CYBERNETICS OR REHABILITATION OR GERIATRICS GERONTOLOGY OR COMPUTER SCIENCE INTERDISCIPLINARY APPLICATIONS) Timespan: 2000-2017. Indexes: SCI-EXPANDED, SSCI, A-HCI, CPCI-S, CPCI-SSH, ESCI.
