# Supplementary material for: Technological State of the Art of Electronic Mental Health Interventions for Major Depressive Disorder: Systematic Literature Review
Source: J Med Internet Res. 2020 Jan 20;22(1):e12599. doi: 10.2196/12599 (PMC6997926; doi:10.2196/12599)
Supplement: Multimedia Appendix 2 [file jmir_v22i1e12599_app2.pdf]

1. Aguilera A, Muñoz RF. Text messaging as an adjunct to CBT in low-income populations: A usability and feasibility pilot study. *Prof Psychol Res Pract*. 2011;42(6):472-478. doi:10.1037/a0025499
2. Agyapong VIO, Milnes J, Farren CK. Supportive text messages for patients with alcohol use disorder and comorbid depression - how useful are they for the patients. *Eur Psychiatry*. 2013;28(March 2015):1. doi:10.1016/s0924-9338(13)75858-9
3. Ahmedani BK, Belville-Robertson T, Hirsch A, Jurayj A. An Online Mental Health and Wellness Intervention Supplementing Standard Care of Depression and Anxiety. *Arch Psychiatr Nurs*. 2016;30(6):666-670. doi:10.1016/j.apnu.2016.03.003
4. Ahmedani BK, Crotty N, Abdulhak MM, Ondersma SJ. Pilot feasibility study of a brief, tailored mobile health intervention for depression among patients with chronic pain. *Behav Med*. 2015;41(1):25-32. doi:10.1080/08964289.2013.867827
5. Aikens JE, Trivedi R, Heapy A, Pfeiffer PN, Piette JD. Potential Impact of Incorporating a Patient-Selected Support Person into mHealth for Depression. *J Gen Intern Med*. 2015;30(6):797-803. doi:10.1007/s11606-015-3208-7
6. Alavi N, Hirji A, Sutton C, Naeem F. Online CBT Is Effective in Overcoming Cultural and Language Barriers in Patients with Depression. *J Psychiatr Pract*. 2016;22(1):2-8. doi:10.1097/PRA.0000000000000119
7. Andersson G, Bergström J, Holländare F, Carlbring P, Kaldo V, Ekselius L. Internet-based self-help for depression: Randomised controlled trial. *Br J Psychiatry*. 2005;187(NOV.):456-461. doi:10.1192/bjp.187.5.456
8. Andersson G, Hesser H, Veilord A, et al. Randomised controlled non-inferiority trial with 3-year follow-up of internet-delivered versus face-to-face group cognitive behavioural therapy for depression. *J Affect Disord*. 2013;151(3):986-994. doi:10.1016/j.jad.2013.08.022
9. Anguera JA, Gunning FM, Areán PA. Improving late life depression and cognitive control through the use of therapeutic video game technology: A proof-of-concept randomized trial. *Depress Anxiety*. 2017;34(6):508-517. doi:10.1002/da.22588
10. Bae J-Y, Lee S-W, Yoon S-H, An K-E. Preliminary evaluation of a web site for depressive symptoms management. *Stud Health Technol Inform*. 2006.
11. Batterham PJ, Neil AL, Bennett K, Griffiths KM, Christensen H. Predictors of adherence among community users of a cognitive behavior therapy website. *Patient Prefer Adherence*. 2008;2(May):97-105. http://www.ncbi.nlm.nih.gov/pubmed/19920949%0Ahttp://www.pubmedcentral.nih.gov/articlerender.fcgi?artid=PMC2770409.
12. Beevers CG, Clasen PC, Enock PM, Schnyer DM. Attention bias modification for major depressive disorder: Effects on attention bias, resting state connectivity, and symptom change. *J Abnorm Psychol*. 2015. doi:10.1037/abn0000049
13. Berger T, Hämmerli K, Gubser N, Andersson G, Caspar F. Internet-Based Treatment of Depression: A Randomized Controlled Trial Comparing Guided with Unguided Self-Help. *Cogn Behav Ther*. 2011;40(4):251-266. doi:10.1080/16506073.2011.616531

14. Berman MI, Buckey JC, Hull JG, et al. Feasibility Study of an Interactive Multimedia Electronic Problem Solving Treatment Program for Depression: A Preliminary Uncontrolled Trial. *Behav Ther*. 2014;45(3):358-375. doi:10.1016/j.beth.2014.02.001
15. Birney AJ, Gunn R, Russell JK, Ary D V. MoodHacker Mobile Web App With Email for Adults to Self-Manage Mild-to-Moderate Depression: Randomized Controlled Trial. *JMIR mHealth uHealth*. 2016;4(1):e8. doi:10.2196/mhealth.4231
16. Blackwell SE, Browning M, Mathews A, et al. Positive imagery-based cognitive bias modification as a web-based treatment tool for depressed adults: A randomized controlled trial. *Clin Psychol Sci*. 2015;3(1):91-111. doi:10.1177/2167702614560746
17. Blackwell SE, Holmes EA. Modifying interpretation and imagination in clinical depression: A single case series using cognitive bias modification. *Appl Cogn Psychol*. 2010;24(3):338-350. doi:10.1002/acp.1680
18. Blom K, Jernelöv S, Kraepelien M, et al. Internet Treatment Addressing either Insomnia or Depression, for Patients with both Diagnoses: A Randomized Trial. *Sleep*. 2015;38(2):267-277. doi:10.5665/sleep.4412
19. Blom MM, Zarit SH, Groot Zwaaftink RBM, Cuijpers P, Pot AM. Effectiveness of an internet intervention for family caregivers of people with dementia: Results of a randomized controlled trial. *PLoS One*. 2015;10(2):1-11. doi:10.1371/journal.pone.0116622
20. Boeschoten RE, Dekker J, Uitdehaag BMJ, et al. Internet-based treatment for depression in multiple sclerosis: A randomized controlled trial. *Mult Scler*. 2017;23(8):1112-1122. doi:10.1177/1352458516671820
21. Boeschoten RE, Nieuwenhuis MM, Van Oppen P, et al. Feasibility and outcome of a web-based self-help intervention for depressive symptoms in patients with multiple sclerosis: A pilot study. *J Neurol Sci*. 2012;315(1-2):104-109. doi:10.1016/j.jns.2011.11.016
22. Bolier L, Haverman M, Kramer J, et al. An internet-Based intervention to promote mental fitness for mildly depressed adults: Randomized controlled trial. *J Med Internet Res*. 2013;15(9):1-16. doi:10.2196/jmir.2603
23. Bond GE, Burr RL, Wolf FM, Feldt K. The effects of a web-based intervention on psychosocial well-being among adults aged 60 and older with diabetes: A randomized trial. *Diabetes Educ*. 2010;36(3):446-456. doi:10.1177/0145721710366758
24. Botella C, Etchemendy E, Castilla D, et al. An e-Health System for the Elderly (Butler Project): A Pilot Study on Acceptance and Satisfaction. *CyberPsychology Behav*. 2009;12(3):255-262. doi:10.1089/cpb.2008.0325
25. Botella C, Mira A, Moragrega I, et al. An internet-based program for depression using activity and physiological sensors: Efficacy, expectations, satisfaction, and ease of use. *Neuropsychiatr Dis Treat*. 2016;12:393-406. doi:10.2147/NDT.S93315
26. Both F, Cuijpers P, Hoogendoorn M, Klein M. TOWARDS FULLY AUTOMATED PSYCHOTHERAPY FOR ADULTS - BAS - Behavioral Activation Scheduling Via Web and Mobile Phone. 2011:375-380. doi:10.5220/0002688503750380
27. Bowie CR, Gupta M, Holshausen K, Jokic R, Best M, Milev R. Cognitive Remediation for Treatment-Resistant Depression: Effects on Cognition and

Functioning and the Role of Online Homework. *J Nerv Ment Dis*. 2013;201(8):680-685. doi:10.1097/NMD.0b013e31829c5030
28. Braithwaite SR, Fincham FD. ePREP: Computer Based Prevention of Relationship Dysfunction, Depression and Anxiety. *J Soc Clin Psychol*. 2007;26(5):609-622. doi:10.1521/jscp.2007.26.5.609
29. Bresó A, Martínez-Miranda J, Fuster-García E, García-Gómez JM. A novel approach to improve the planning of adaptive and interactive sessions for the treatment of Major Depression. *Int J Hum Comput Stud*. 2016;87:80-91. doi:10.1016/j.ijhcs.2015.11.003
30. Browning M, Holmes EA, Charles M, Cowen PJ, Harmer CJ. Using attentional bias modification as a cognitive vaccine against depression. *Biol Psychiatry*. 2012;72(7):572-579. doi:10.1016/j.biopsych.2012.04.014
31. Buhrman M, Syk M, Burvall O, Hartig T, Gordh T, Andersson G. Individualized guided internet-delivered cognitive-behavior therapy for chronic pain patients with comorbid depression and anxiety: A randomized controlled trial. *Clin J Pain*. 2015;31(6):504-516. doi:10.1097/AJP.0000000000000176
32. Bunge EL, Williamson RE, Cano M, Leykin Y, Muñoz RF. Mood management effects of brief unsupported internet interventions. *Internet Interv*. 2016;5:36-43. doi:10.1016/j.invent.2016.06.001
33. Buntrock C, Ebert DD, Lehr D, et al. Effect of a web-based guided self-help intervention for prevention of major depression in adults with subthreshold depression a randomized clinical trial. *JAMA - J Am Med Assoc*. 2016;315(17):1854-1863. doi:10.1001/jama.2016.4326
34. Buntrock C, Ebert D, Lehr D, et al. Effectiveness of a Web-Based Cognitive Behavioural Intervention for Subthreshold Depression: Pragmatic Randomised Controlled Trial. *Psychother Psychosom*. 2015;84(6):348-358. doi:10.1159/000438673
35. Burns MN, Begale M, Duffecy J, et al. Harnessing context sensing to develop a mobile intervention for depression. *J Med Internet Res*. 2011;13(3):1-19. doi:10.2196/jmir.1838
36. Burton C, Szentagotai Tatar A, McKinstry B, et al. Pilot randomised controlled trial of Help4Mood, an embodied virtual agent-based system to support treatment of depression. *J Telemed Telecare*. 2016;22(6):348-355. doi:10.1177/1357633x15609793
37. Bush NE, Ouellette G, Kinn J. Utility of the T2 Mood Tracker Mobile Application Among Army Warrior Transition Unit Service Members. *Mil Med*. 2014;179(12):1453-1457. doi:10.7205/milmed-d-14-00271
38. Calkins AW, McMorran KE, Siegle GJ, Otto MW. The Effects of Computerized Cognitive Control Training on Community Adults with Depressed Mood. *Behav Cogn Psychother*. 2015;43(5):578-589. doi:10.1017/s1352465814000046
39. Carlbring P, Hägglund M, Luthström A, et al. Internet-based behavioral activation and acceptance-based treatment for depression: A randomized controlled trial. *J Affect Disord*. 2013;148(2-3):331-337. doi:10.1016/j.jad.2012.12.020
40. Cartreine JA, Locke SE, Buckey JC, Sandoval L, Hegel MT. Electronic problem-solving treatment: Description and pilot study of an interactive media treatment for depression. *J Med Internet Res*. 2012;14(5):1-14. doi:10.2196/resprot.1925

41. Cassimatis M, Kavanagh DJ, Hills AP, et al. Development of the OnTrack Diabetes Program. *JMIR Res Protoc*. 2015;4(2):e24. doi:10.2196/resprot.2823
42. Cassimatis M, Kavanagh DJ, Hills AP, et al. The OnTrack Diabetes Web-Based Program for Type 2 Diabetes and Dysphoria Self-Management: A Randomized Controlled Trial Protocol. *JMIR Res Protoc*. 2015;4(3):e97. doi:10.2196/resprot.2813
43. Cavanagh K, Shapiro DA, Van Den Berg S, Swain S, Barkham M, Proudfoot J. The effectiveness of computerized cognitive behavioural therapy in routine care. *Br J Clin Psychol*. 2006;45(4):499-514. doi:10.1348/014466505X84782
44. Cavanagh K, Shapiro DA, van Den Berg S, Swain S, Barkham M, Proudfoot J. The acceptability of computer-aided cognitive behavioural therapy: A pragmatic study. *Cogn Behav Ther*. 2009;38(4):235-246. doi:10.1080/16506070802561256
45. Cavanagh K, Strauss C, Cicconi F, Griffiths N, Wyper A, Jones F. A randomised controlled trial of a brief online mindfulness-based intervention. *Behav Res Ther*. 2013;51(9):573-578. doi:10.1016/j.brat.2013.06.003
46. Chan R, Dear BF, Titov N, Chow J, Suranyi M. Examining internet-delivered cognitive behaviour therapy for patients with chronic kidney disease on haemodialysis: A feasibility open trial. *J Psychosom Res*. 2016;89:78-84. doi:10.1016/j.jpsychores.2016.08.012
47. Choi I, Zou J, Titov N, et al. Culturally attuned internet treatment for depression amongst Chinese Australians: A randomised controlled trial. *J Affect Disord*. 2012;136(3):459-468. doi:10.1016/j.jad.2011.11.003
48. Christensen H, Batterham PJ, Gosling JA, et al. Effectiveness of an online insomnia program (SHUTi) for prevention of depressive episodes (the GoodNight Study): A randomised controlled trial. *The Lancet Psychiatry*. 2016;3(4):333-341. doi:10.1016/S2215-0366(15)00536-2
49. Christensen H, Griffiths KM, Mackinnon AJ, Brittliffe K. Online randomized controlled trial of brief and full cognitive behaviour therapy for depression. *Psychol Med*. 2006;36(12):1737-1746. doi:10.1017/S0033291706008695
50. Christensen H, Griffiths KM, Jorm AF. Delivering interventions for depression by using the internet: randomised controlled trial. *BMJ*. 2004;328(7434):265. doi:10.1136/bmj.37945.566632.EE
51. Christensen H, Griffiths KM, Korten AE, Brittliffe K, Groves C. A comparison of changes in anxiety and depression symptoms of spontaneous users and trial participants of a cognitive behavior therapy website. *J Med Internet Res*. 2004;6(4):e46. doi:10.2196/jmir.6.4.e46
52. Christensen H, Griffiths KM, Korten A. Web-based cognitive behavior therapy: Analysis of site usage and changes in depression and anxiety scores. *J Med Internet Res*. 2002;4(1):29-40. doi:10.2196/jmir.4.1.e3
53. Clarke G, Eubanks D, Reid E, et al. Overcoming depression on the internet (ODIN) (2): A randomized trial of a self-help depression skills program with reminders. *J Med Internet Res*. 2005;7(2):1-12. doi:10.2196/jmir.7.2.e16
54. Clarke G, Kelleher C, Hornbrook M, DeBar L, Dickerson J, Gullion C. Randomized effectiveness trial of an internet, pure self-help, cognitive behavioral intervention for depressive symptoms in young adults. *Cogn Behav Ther*. 2009;38(4):222-234. doi:10.1080/16506070802675353

55. Clarke G, Reid E, Eubanks D, et al. Overcoming Depression on the Internet (ODIN): A randomized controlledtrialof an Internet depression skills intervention program. *J Med Internet Res*. 2002;4(3):5-17. doi:10.2196/jmir.4.3.e14
56. Clarke J, Proudfoot J, Ma H. Mobile Phone and Web-based Cognitive Behavior Therapy for Depressive Symptoms and Mental Health Comorbidities in People Living With Diabetes: Results of a Feasibility Study. *JMIR Ment Heal*. 2016;3(2):e23. doi:10.2196/mental.5131
57. Cohn MA, Pietrucha ME, Saslow LR, Hult JR, Moskowitz JT. An online positive affect skills intervention reduces depression in adults with type 2 diabetes. *J Posit Psychol*. 2014. doi:10.1080/17439760.2014.920410
58. Cooper CL, Hind D, Parry GD, et al. Computerised cognitive behavioural therapy for the treatment of depression in people with multiple sclerosis: External pilot trial. *Trials*. 2011;12:1-14. doi:10.1186/1745-6215-12-259
59. Cukrowicz KC, Joiner TE. Computer-based intervention for anxious and depressive symptoms in a non-clinical population. *Cognit Ther Res*. 2007;31(5):677-693. doi:10.1007/s10608-006-9094-x
60. Currie SL, Mcgrath PJ, Day V. Development and usability of an online CBT program for symptoms of moderate depression, anxiety, and stress in post-secondary students. *Comput Human Behav*. 2010;26(6):1419-1426. doi:10.1016/j.chb.2010.04.020
61. Day V, McGrath PJ, Wojtowicz M. Internet-based guided self-help for university students with anxiety, depression and stress: A randomized controlled clinical trial. *Behav Res Ther*. 2013;51(7):344-351. doi:10.1016/j.brat.2013.03.003
62. De Graaf LE, Gerhards SAH, Arntz A, et al. Clinical effectiveness of online computerised cognitive-behavioural therapy without support for depression in primary care: Randomised trial. *Br J Psychiatry*. 2009;195(1):73-80. doi:10.1192/bjp.bp.108.054429
63. Deady M, Kay-Lambkin F, Teesson M, Mills K. Developing an integrated, Internet-based self-help programme for young people with depression and alcohol use problems. *Internet Interv*. 2014;1(3):118-131. doi:10.1016/j.invent.2014.06.004
64. Deady M, Mills KL, Teesson M, Kay-Lambkin F. An online intervention for co-occurring depression and problematic alcohol use in young people: Primary outcomes from a randomized controlled trial. *J Med Internet Res*. 2016;18(3):1-12. doi:10.2196/jmir.5178
65. Dear BF, Gandy M, Karin E, et al. The Pain Course. *Pain*. 2015;156(10):1920-1935. doi:10.1097/j.pain.0000000000000251
66. Dear BF, Titov N, Perry KN, et al. The Pain Course: A randomised controlled trial of a clinician-guided Internet-delivered cognitive behaviour therapy program for managing chronic pain and emotional well-being. *Pain*. 2013;154(6):942-950. doi:10.1016/j.pain.2013.03.005
67. Dear BF, Titov N, Schwencke G, et al. An open trial of a brief transdiagnostic internet treatment for anxiety and depression. *Behav Res Ther*. 2011;49(12):830-837. doi:10.1016/j.brat.2011.09.007
68. Dear BF, Zou J, Titov N, et al. Internet-delivered cognitive behavioural therapy for depression: A feasibility open trial for older adults. *Aust N Z J Psychiatry*. 2013;47(2):169-176. doi:10.1177/0004867412466154

69. Dimidjian S, Beck A, Felder JN, Boggs JM, Gallop R, Segal Z V. Web-based Mindfulness-based Cognitive Therapy for reducing residual depressive symptoms: An open trial and quasi-experimental comparison to propensity score matched controls. *Behav Res Ther*. 2014;63:83-89. doi:10.1016/j.brat.2014.09.004
70. Dobbin A, Maxwell M, Elton R. A benchmarked feasibility study of a self-hypnosis treatment for depression in primary care. *Int J Clin Exp Hypn*. 2009;57(3):293-318. doi:10.1080/00207140902881221
71. Doherty G, Coyle D, Sharry J. Engagement with online mental health interventions: An exploratory clinical study of a treatment for depression. In: *Proceedings of the SIGCHI Conference on Human Factors in Computing Systems*. ; 2012:1421. doi:10.1145/2207676.2208602
72. Donker T, Bennett K, Bennett A, et al. Internet-delivered interpersonal psychotherapy versus internet-delivered cognitive behavioral therapy for adults with depressive symptoms: Randomized controlled noninferiority trial. *J Med Internet Res*. 2013;15(5):1-16. doi:10.2196/jmir.2307
73. Drake G, Csipke E, Wykes T. Assessing your mood online: Acceptability and use of Moodscope. *Psychol Med*. 2013;43(7):1455-1464. doi:10.1017/S0033291712002280
74. Drozd F, Skeie LG, Kraft P, Kvale D. A web-based intervention trial for depressive symptoms and subjective well-being in patients with chronic HIV infection. *AIDS Care - Psychol Socio-Medical Asp AIDS/HIV*. 2014;26(9):1080-1089. doi:10.1080/09540121.2013.869541
75. Ebert DD, Lehr D, Boß L, et al. Efficacy of an internet-based problem-solving training for teachers: Results of a randomized controlled trial. *Scand J Work Environ Heal*. 2014;40(6):582-596. doi:10.5271/sjweh.3449
76. Eisma MC, Boelen PA, van den Bout J, et al. Internet-Based Exposure and Behavioral Activation for Complicated Grief and Rumination: A Randomized Controlled Trial. *Behav Ther*. 2015;46(6):729-748. doi:10.1016/j.beth.2015.05.007
77. Espinosa HD, Carrasco Á, Moessner M, et al. Acceptability Study of "Ascenso": An Online Program for Monitoring and Supporting Patients with Depression in Chile. *Telemed e-Health*. 2016;22(7):577-583. doi:10.1089/tmj.2015.0124
78. Falconer CJ, Rovira A, King JA, et al. Embodying self-compassion within virtual reality and its effects on patients with depression. *BJPsych Open*. 2016;2(1):74-80. doi:10.1192/bjpo.bp.115.002147
79. Farrer L, Christensen H, Griffiths KM, Mackinnon A. Internet-based CBT for depression with and without telephone tracking in a national helpline: Randomised controlled trial. *PLoS One*. 2011;6(11). doi:10.1371/journal.pone.0028099
80. Fischer A, Schröder J, Vettorazzi E, et al. An online programme to reduce depression in patients with multiple sclerosis: A randomised controlled trial. *The Lancet Psychiatry*. 2015;2(3):217-223. doi:10.1016/S2215-0366(14)00049-2
81. Fledderus M, Bohlmeijer ET, Fox JP, Schreurs KMG, Spinhoven P. The role of psychological flexibility in a self-help acceptance and commitment therapy intervention for psychological distress in a randomized controlled trial. *Behav Res Ther*. 2013;51(3):142-151. doi:10.1016/j.brat.2012.11.007

82. Gandy M, Karin E, Fogliati VJ, McDonald S, Titov N, Dear BF. A feasibility trial of an Internet-delivered and transdiagnostic cognitive behavioral therapy treatment program for anxiety, depression, and disability among adults with epilepsy. *Epilepsia*. 2016;57(11):1887-1896. doi:10.1111/epi.13569
83. Geisner IM, Varvil-Weld L, Mittmann AJ, Mallett K, Turrisi R. Brief web-based intervention for college students with comorbid risky alcohol use and depressed mood: Does it work and for whom? *Addict Behav*. 2015;42:36-43. doi:10.1016/j.addbeh.2014.10.030
84. Geraedts AS, Kleiboer AM, Twisk J, Wiezer NM, Van Mechelen W, Cuijpers P. Long-term results of a web-based guided self-help intervention for employees with depressive symptoms: Randomized controlled trial. *J Med Internet Res*. 2014;16(7):1-17. doi:10.2196/jmir.3539
85. Gilbody S, Littlewood E, Hewitt C, et al. Computerised cognitive behaviour therapy (cCBT) as treatment for depression in primary care (REEACT trial): Large scale pragmatic randomised controlled trial. *BMJ*. 2015;351:1-13. doi:10.1136/bmj.h5627
86. Glozier N, Christensen H, Naismith S, et al. Internet-Delivered Cognitive Behavioural Therapy for Adults with Mild to Moderate Depression and High Cardiovascular Disease Risks: A Randomised Attention-Controlled Trial. *PLoS One*. 2013;8(3):1-8. doi:10.1371/journal.pone.0059139
87. González-Robles A, Mira A, Díaz A, et al. Opinion of the Patients About an Internet-Based Psychological Treatment Protocol. In: *Lecture Notes in Computer Science (Including Subseries Lecture Notes in Artificial Intelligence and Lecture Notes in Bioinformatics)*. ; 2015:460-466. doi:10.1007/978-3-319-26401-1_43
88. Griffiths KM, Bennett K, Walker J, Goldsmid S, Bennett A. Effectiveness of MH-Guru, a brief online mental health program for the workplace: A randomised controlled trial. *Internet Interv*. 2016;6:29-39. doi:10.1016/j.invent.2016.09.004
89. Griffiths KM, Mackinnon AJ, Crisp DA, Christensen H, Bennett K, Farrer L. The Effectiveness of an Online Support Group for Members of the Community with Depression: A Randomised Controlled Trial. *PLoS One*. 2012;7(12). doi:10.1371/journal.pone.0053244
90. Grime PR. Computerized cognitive behavioural therapy at work: A randomized controlled trial in employees with recent stress-related absenteeism. *Occup Med (Chic Ill)*. 2004;54(5):353-359. doi:10.1093/occmed/kqh077
91. Hadjistavropoulos HD, Nugent MM, Alberts NM, Staples L, Dear BF, Titov N. Transdiagnostic Internet-delivered cognitive behaviour therapy in Canada: An open trial comparing results of a specialized online clinic and nonspecialized community clinics. *J Anxiety Disord*. 2016;42:19-29. doi:10.1016/j.janxdis.2016.05.006
92. Hadjistavropoulos HD, Pugh NE, Nugent MM, et al. Therapist-assisted Internet-delivered cognitive behavior therapy for depression and anxiety: Translating evidence into clinical practice. *J Anxiety Disord*. 2014;28(8):884-893. doi:10.1016/j.janxdis.2014.09.018
93. Hallgren M, Helgadóttir B, Herring MP, et al. Exercise and internet-based cognitive-behavioural therapy for depression: Multicentre randomized controlled trial with 12-month follow-up. *Br J Psychiatry*. 2016;209(5):414-420. doi:10.1192/bjp.bp.115.177576

94. Hetrick SE, Goodall J, Yuen HP, et al. Comprehensive online self- monitoring to support clinicians manage risk of suicide in youth depression: A pilot study. *Crisis*. 2017;38(3):147-157. doi:10.1027/0227-5910/a000422
95. Hinkle JF. The Stress Gym: An Online Intervention to Improve Stress and Depressive Symptoms in Adults. *Issues Ment Health Nurs*. 2015;36(11):870-876. doi:10.3109/01612840.2015.1074768
96. Høifødt RS, Lillevoll KR, Griffiths KM, et al. The clinical effectiveness of web-based cognitive behavioral therapy with face-to-face therapist support for depressed primary care patients: Randomized controlled trial. *J Med Internet Res*. 2013;15(8). doi:10.2196/jmir.2714
97. Høifødt RS, Mittner M, Lillevoll K, et al. Predictors of response to web-based cognitive behavioral therapy with high-intensity face-to-face therapist guidance for depression: A Bayesian analysis. *J Med Internet Res*. 2015;17(9):e197. doi:10.2196/jmir.4351
98. Holländare F, Johnsson S, Randestad M, et al. Randomized trial of Internet-based relapse prevention for partially remitted depression. *Acta Psychiatr Scand*. 2011;124(4):285-294. doi:10.1111/j.1600-0447.2011.01698.x
99. Hoorelbeke K, Faelens L, Behiels J, Koster EHW. Internet-delivered cognitive control training as a preventive intervention for remitted depressed patients: Protocol for a randomized controlled trial. *BMC Psychiatry*. 2015;15(1):125. doi:10.1186/s12888-015-0511-0
100. Hunkeler EM, Hargreaves WA, Fireman B, et al. A Web-Delivered Care Management and Patient Self-Management Program for Recurrent Depression: A Randomized Trial. *Psychiatr Serv*. 2013;63(11):1063-1071. doi:10.1176/appi.ps.005332011
101. Imamura K, Kawakami N, Furukawa TA, et al. Effects of an internet-based cognitive behavioral therapy (iCBT) program in manga format on improving subthreshold depressive symptoms among healthy workers: A randomized controlled trial. *PLoS One*. 2014;9(5):e97167. doi:10.1371/journal.pone.0097167
102. Imamura K, Kawakami N, Tsuno K, Tsuchiya M, Shimada K, Namba K. Effects of web-based stress and depression literacy intervention on improving symptoms and knowledge of depression among workers: A randomized controlled trial. *J Affect Disord*. 2016;203:30-37. doi:10.1016/j.jad.2016.05.045
103. Jakobsen H, Andersson G, Havik OE, Nordgreen T. Guided Internet-based cognitive behavioral therapy for mild and moderate depression: A benchmarking study. *Internet Interv*. 2017;7:1-8. doi:10.1016/j.invent.2016.11.002
104. Janevic MR, Aruquipa Yujra AC, Marinec N, et al. Feasibility of an interactive voice response system for monitoring depressive symptoms in a lower-middle income Latin American country. *Int J Ment Health Syst*. 2016;10(1):1-11. doi:10.1186/s13033-016-0093-3
105. Johansson R, Björklund M, Hornborg C, et al. Affect-focused psychodynamic psychotherapy for depression and anxiety through the Internet: a randomized controlled trial. *PeerJ*. 2013;1:e102. doi:10.7717/peerj.102
106. Johansson R, Ekbladh S, Hebert A, et al. Psychodynamic guided self-help for adult depression through the internet: A randomised controlled trial. *PLoS One*. 2012;7(5). doi:10.1371/journal.pone.0038021

107. Johansson R, Nyblom A, Carlbring P, Cuijpers P, Andersson G. Choosing between Internet-based psychodynamic versus cognitive behavioral therapy for depression: A pilot preference study. *BMC Psychiatry*. 2013. doi:10.1186/1471-244X-13-268
108. Johansson R, Sjöberg E, Sjögren M, et al. Tailored vs. Standardized internet-based cognitive behavior therapy for depression and comorbid symptoms: A randomized controlled trial. *PLoS One*. 2012;7(5):1-9. doi:10.1371/journal.pone.0036905
109. Johnston L, Dear BF, Gandy M, et al. Exploring the efficacy and acceptability of Internet-delivered cognitive behavioural therapy for young adults with anxiety and depression: An open trial. *Aust N Z J Psychiatry*. 2014;48(9):819-827. doi:10.1177/0004867414527524
110. Joutsenniemi K. E-mail-based Exercises in Happiness, Physical Activity and Readings: A Randomized Trial on 3274 Finns. *J Psychiatry*. 2015;17(5). doi:10.4172/psychiatry.1000140
111. Kawai K, Yamazaki Y, Nakayama K. Process Evaluation of a Web-based Stress Management Program to Promote Psychological Well-being in a Sample of White-collar Workers in Japan. *Ind Health*. 2010. doi:10.2486/indhealth.48.265
112. Kay-Lambkin FJ, Baker AL, Kelly B, Lewin TJ. Clinician-assisted computerised versus therapist-delivered treatment for depressive and addictive disorders: A randomised controlled trial. *Med J Aust*. 2011. doi:10.5694/j.1326-5377.2011.tb03265.x
113. Kay-Lambkin FJ, Baker AL, Lewin TJ, Carr VJ. Computer-based psychological treatment for comorbid depression and problematic alcohol and/or cannabis use: A randomized controlled trial of clinical efficacy. *Addiction*. 2009;104(3):378-388. doi:10.1111/j.1360-0443.2008.02444.x
114. Kayrouz R, Dear BF, Karin E, Fogliati VJ, Titov N. A pilot study of a clinician-guided internet-delivered cognitive behavioural therapy for anxiety and depression among Arabs in Australia, presented in both English and Arabic languages. *Internet Interv*. 2016;5:5-11. doi:10.1016/j.invent.2016.06.002
115. Kayrouz R, Dear BF, Karin E, et al. A pilot study of self-guided internet-delivered cognitive behavioural therapy for anxiety and depression among Arabs. *Internet Interv*. 2016;3:18-24. doi:10.1016/j.invent.2015.10.005
116. Kelders SM, Bohlmeijer ET, Pots WTM, van Gemert-Pijnen JEWC. Comparing human and automated support for depression: Fractional factorial randomized controlled trial. *Behav Res Ther*. 2015;72:72-80. doi:10.1016/j.brat.2015.06.014
117. Kenter RMF, Cuijpers P, Beekman A, van Straten A. Effectiveness of a Web-Based Guided Self-help Intervention for Outpatients With a Depressive Disorder: Short-term Results From a Randomized Controlled Trial. *J Med Internet Res*. 2016;18(3):e80. doi:10.2196/jmir.4861
118. Kenter R, Warmerdam L, Brouwer-Dudokdewit C, Cuijpers P, van Straten A. Guided online treatment in routine mental health care: An observational study on uptake, drop-out and effects. *BMC Psychiatry*. 2013;13(1):43. doi:10.1186/1471-244X-13-43
119. Killen A, Macaskill A. Using a Gratitude Intervention to Enhance Well-Being in Older Adults. *J Happiness Stud*. 2015;16(4):947-964. doi:10.1007/s10902-014-9542-3

120. Kirkpatrick T, Manoukian L, Dear BF, Johnston L, Titov N. A feasibility open trial of internet-delivered cognitive-behavioural therapy (iCBT) among consumers of a non-governmental mental health organisation with anxiety. *PeerJ*. 2013. doi:10.7717/peerj.210
121. Kleiboer A, Donker T, Seekles W, van Straten A, Riper H, Cuijpers P. A randomized controlled trial on the role of support in Internet-based problem solving therapy for depression and anxiety. *Behav Res Ther*. 2015;72(July):63-71. doi:10.1016/j.brat.2015.06.013
122. Klein JP, Berger T, Schr’der J, et al. Effects of a psychological internet intervention in the treatment of mild to moderate depressive symptoms: Results of the evident study, a randomized controlled trial. *Psychother Psychosom*. 2016;85(4):218-228. doi:10.1159/000445355
123. Kok G, Burger H, Riper H, et al. The three-month effect of mobile internet-based cognitive therapy on the course of depressive symptoms in remitted recurrently depressed patients: Results of a randomized controlled trial. *Psychother Psychosom*. 2015;84(2):90-99. doi:10.1159/000369469
124. Kooistra LC, Ruwaard J, Wiersma JE, et al. Development and initial evaluation of blended cognitive behavioural treatment for major depression in routine specialized mental health care. *Internet Interv*. 2016;4:61-71. doi:10.1016/j.invent.2016.01.003
125. Kordy H, Wolf M, Aulich K, et al. Internet-Delivered Disease Management for Recurrent Depression: A Multicenter Randomized Controlled Trial. *Psychother Psychosom*. 2016;85(2):91-98. doi:10.1159/000441951
126. Kraaij V, van Emmerik A, Garnefski N, et al. Effects of a cognitive behavioral self-help program and a computerized structured writing intervention on depressed mood for HIV-infected people: A pilot randomized controlled trial. *Patient Educ Couns*. 2010;80(2):200-204. doi:10.1016/j.pec.2009.08.014
127. Kraft S, Wolf M, Klein T, Becker T, Bauer S, Puschner B. Text Message Feedback to Support Mindfulness Practice in People With Depressive Symptoms: A Pilot Randomized Controlled Trial. *JMIR mHealth uHealth*. 2017;5(5):e59. doi:10.2196/mhealth.7095
128. Kroenke K, Theobald D, Wu J, et al. Effect of Telecare Management on Pain and Depression in Patients With Cancer. *JAMA*. 2010;304(2):163. doi:10.1001/jama.2010.944
129. Krusche A, Cyhlarova E, Williams JMG. Mindfulness online: An evaluation of the feasibility of a web-based mindfulness course for stress, anxiety and depression. *BMJ Open*. 2013;3(11). doi:10.1136/bmjopen-2013-003498
130. Lai T-Y, Larson EL, Rockoff ML, Bakken S. User Acceptance of HIV TIDES--Tailored Interventions for Management of Depressive Symptoms in Persons Living with HIV/AIDS. *J Am Med Informatics Assoc*. 2008;15(2):217-226. doi:10.1197/jamia.M2481
131. Lai T-Y. Iterative refinement of a tailored system for self-care management of depressive symptoms in people living with HIV/AIDS through heuristic evaluation and end user testing. *Int J Med Inform*. 2007;76:S317-S324. doi:10.1016/j.ijmedinf.2007.05.007

132. Lai T-Y, Bakken S. Heuristic evaluation of HIV-TIDES - Tailored Interventions for management of DEpressive Symptoms in HIV-infected individuals. *AMIA . Annu Symp proceedings AMIA Symp*. 2006:996.
133. Lang TJ, Blackwell SE, Harmer CJ, Davison P, Holmes EA. Cognitive Bias Modification Using Mental Imagery for Depression: Developing a Novel Computerized Intervention to Change Negative Thinking Styles. *Eur J Pers*. 2012;26(2):145-157. doi:10.1002/per.855
134. Lappalainen P, Granlund A, Siltanen S, et al. ACT Internet-based vs face-to-face? A randomized controlled trial of two ways to deliver Acceptance and Commitment Therapy for depressive symptoms: An 18-month follow-up. *Behav Res Ther*. 2014;61(July 2015):43-54. doi:10.1016/j.brat.2014.07.006
135. Lappalainen P, Langrial S, Oinas-Kukkonen H, Tolvanen A, Lappalainen R. Web-Based Acceptance and Commitment Therapy for Depressive Symptoms With Minimal Support: A Randomized Controlled Trial. *Behav Modif*. 2015;39(6):805-834. doi:10.1177/0145445515598142
136. Lara MA, Tiburcio M, Aguilar Abrego A, Sánchez-Solís A. A four-year experience with a Web-based self-help intervention for depressive symptoms in Mexico. *Rev Panam Salud Publica*. 2014;35(5-6):399-406. http://www.ncbi.nlm.nih.gov/pubmed/25211568.
137. Learmonth D, Rai S. Taking computerized CBT beyond primary care. *Br J Clin Psychol*. 2008;47(1):111-118. doi:10.1348/014466507X248599
138. Lee JS, Mathews A, Shergill S, Yiu Chan DK, Majeed N, Yiend J. How can we enhance cognitive bias modification techniques? The effects of prospective cognition. *J Behav Ther Exp Psychiatry*. 2015;49(March):120-127. doi:10.1016/j.jbtep.2015.03.007
139. Lee SW, Kim I, Yoo J, Park S, Jeong B, Cha M. Insights from an expressive writing intervention on Facebook to help alleviate depressive symptoms. *Comput Human Behav*. 2016;62:613-619. doi:10.1016/j.chb.2016.04.034
140. Lemma A, Fonagy P. Feasibility study of a psychodynamic online group intervention for depression. *Psychoanal Psychol*. 2013;30(3):367-380. doi:10.1037/a0033239
141. Levin W, Campbell DR, McGovern KB, et al. A computer-assisted depression intervention in primary care. *Psychol Med*. 2011;41(7):1373-1383. doi:10.1017/S0033291710001935
142. Leykin Y, Muñoz RF, Contreras O, Latham MD. Results from a trial of an unsupported internet intervention for depressive symptoms. *Internet Interv*. 2014;1(4):175-181. doi:10.1016/j.invent.2014.09.002
143. Lillevoll KR, Vangberg HCB, Griffiths KM, Waterloo K, Eisemann MR. Uptake and adherence of a self-directed internet-based mental health intervention with tailored e-mail reminders in senior high schools in Norway. *BMC Psychiatry*. 2014. doi:10.1186/1471-244X-14-14
144. Lillevoll KR, Wilhelmsen M, Kolstrup N, et al. Patients' experiences of helpfulness in guided internet-based treatment for depression: Qualitative study of integrated therapeutic dimensions. *J Med Internet Res*. 2013;15(6):1-12. doi:10.2196/jmir.2531

145. Lin MF, Moyle W, Chang HJ, Chou MH, Hsu MC. Effect of an interactive computerized psycho-education system on patients suffering from depression. *J Clin Nurs*. 2008;17(5):667-676. doi:10.1111/j.1365-2702.2007.02085.x
146. Lintvedt OK, Griffiths KM, Sørensen K, et al. Evaluating the effectiveness and efficacy of unguided internet-based self-help intervention for the prevention of depression: A randomized controlled trial. *Clin Psychol Psychother*. 2013;20(1):10-27. doi:10.1002/cpp.770
147. Littlewood E, Duarte A, Hewitt C, et al. A randomised controlled trial of computerised cognitive behaviour therapy for the treatment of depression in primary care: The Randomised Evaluation of the Effectiveness and Acceptability of Computerised Therapy (REEACT) trial. *Health Technol Assess (Rockv)*. 2015;19(101). doi:10.3310/hta191010
148. Løventoft PK, Nørregaard LB, Frøkjær E. Designing daybuilder: An Experimental App to Support People with Depression. *Proc 12th Particip Des Conf Explor Pap Work Descr Ind Cases - Vol 2 - PDC '12*. 2012;(May 2014):1. doi:10.1145/2348144.2348146
149. Lucassen MFG, Merry SN, Hatcher S, Frampton CMA. Rainbow SPARX: A novel approach to addressing depression in sexual minority youth. *Cogn Behav Pract*. 2015;22(2):203-216. doi:10.1016/j.cbpra.2013.12.008
150. Lundgren JG, Dahlström Ö, Andersson G, Jaarsma T, Kärner Köhler A, Johansson P. The Effect of Guided Web-Based Cognitive Behavioral Therapy on Patients With Depressive Symptoms and Heart Failure: A Pilot Randomized Controlled Trial. *J Med Internet Res*. 2016;18(8):e194. doi:10.2196/jmir.5556
151. Lundgren J, Andersson G, Dahlström Ö, Jaarsma T, Köhler AK, Johansson P. Internet-based cognitive behavior therapy for patients with heart failure and depressive symptoms: A proof of concept study. *Patient Educ Couns*. 2015;98(8):935-942. doi:10.1016/j.pec.2015.04.013
152. Ly KH, Topooco N, Cederlund H, et al. Smartphone-supported versus full behavioural activation for depression: A randomised controlled trial. *PLoS One*. 2015;10(5):1-16. doi:10.1371/journal.pone.0126559
153. Ly KH, Trüschel A, Jarl L, et al. Behavioural activation versus mindfulness-based guided self-help treatment administered through a smartphone application: a randomised controlled trial. *BMJ Open*. 2014;4(1):e003440. doi:10.1136/bmjopen-2013-003440
154. Månsson KNT, Ruiz ES, Gervind E, Dahlin M, Andersson G. Development and initial evaluation of an internet-based support system for face-to-face cognitive behavior therapy: A proof of concept study. *J Med Internet Res*. 2013;15(12). doi:10.2196/jmir.3031
155. McCombie A, Gearry R, Andrews J, Mulder R, Mikocka-Walus A. Does Computerized Cognitive Behavioral Therapy Help People with Inflammatory Bowel Disease? A Randomized Controlled Trial. *Inflamm Bowel Dis*. 2016;22(1):171-181. doi:10.1097/MIB.0000000000000567
156. McMurchie W, Macleod F, Power K, Laidlaw K, Prentice N. Computerised cognitive behavioural therapy for depression and anxiety with older people: a pilot study to examine patient acceptability and treatment outcome. *Int J Geriatr Psychiatry*. 2013:n/a-n/a. doi:10.1002/gps.3935

157. Meglic M, Furlan M, Kuzmanic M, et al. Feasibility of an eHealth service to support collaborative depression care: Results of a pilot study. *J Med Internet Res*. 2010;12(5):1-11. doi:10.2196/jmir.1510
158. Meiser B, Peate M, Levitan C, et al. A Psycho-Educational Intervention for People with a Family History of Depression: Pilot Results. *J Genet Couns*. 2017;26(2):312-321. doi:10.1007/s10897-016-0011-5
159. Melnyk BM, Amaya M, Szalacha LA, Hoying J, Taylor T, Bowersox K. Feasibility, Acceptability, and Preliminary Effects of the COPE Online Cognitive-Behavioral Skill-Building Program on Mental Health Outcomes and Academic Performance in Freshmen College Students: A Randomized Controlled Pilot Study. *J Child Adolesc Psychiatr Nurs*. 2015;28(3):147-154. doi:10.1111/jcap.12119
160. Merry SN, Stasiak K, Shepherd M, Frampton C, Fleming T, Lucassen MFG. The effectiveness of SPARX, a computerised self help intervention for adolescents seeking help for depression: Randomised controlled non-inferiority trial. *BMJ*. 2012;344(7857):1-16. doi:10.1136/bmj.e2598
161. Mewton L, Hobbs MJ, Sunderland M, Newby J, Andrews G. Reductions in the internalising construct following internet-delivered treatment for anxiety and depression in primary care. *Behav Res Ther*. 2014;63(March 2016):132-138. doi:10.1016/j.brat.2014.10.001
162. Mewton L, Andrews G. Cognitive behaviour therapy via the internet for depression: A useful strategy to reduce suicidal ideation. *J Affect Disord*. 2015;170:78-84. doi:10.1016/j.jad.2014.08.038
163. Meyer B, Berger T, Caspar F, Beevers CG, Andersson G, Weiss. Effectiveness of a novel integrative online treatment for depression (Deprexis): Randomized field trial. 2009:1-8.
164. Mira A, Bretón-López J, García-Palacios A, Baños RM, Botella C. *Pervasive Computing Paradigms for Mental Health*. Vol 604. (Serino S, Matic A, Giakoumis D, Lopez G, Cipresso P, eds.). Cham: Springer International Publishing; 2016. doi:10.1007/978-3-319-32270-4
165. Möbius M, Tendolkar I, Lohner V, Baltussen M, Becker ES. Refilling the half-empty glass - Investigating the potential role of the Interpretation Modification Paradigm for Depression (IMP-D). *J Behav Ther Exp Psychiatry*. 2015;49:37-43. doi:10.1016/j.jbtep.2015.03.002
166. Mogoaşe C, Brăilean A, David D. Can concreteness training alone reduce depressive symptoms? A randomized pilot study using an internet-delivered protocol. *Cognit Ther Res*. 2013;37(4):704-712. doi:10.1007/s10608-012-9514-z
167. Mohr DC, Duffecy J, Ho J, et al. A Randomized Controlled Trial Evaluating a Manualized TeleCoaching Protocol for Improving Adherence to a Web-Based Intervention for the Treatment of Depression. *PLoS One*. 2013;8(8). doi:10.1371/journal.pone.0070086
168. Mohr DC, Duffecy J, Jin L, et al. Multimodal e-mental health treatment for depression: A feasibility trial. *J Med Internet Res*. 2010;12(5):1-12. doi:10.2196/jmir.1370
169. Montero-Marín J, Araya R, Pérez-Yus MC, et al. An internet-based intervention for depression in primary care in Spain: A randomized controlled trial. *J Med Internet Res*. 2016;18(8):1-12. doi:10.2196/jmir.5695

170. Morgan AJ, Jorm AF, Mackinnon AJ. Email-based promotion of self-help for subthreshold depression: Mood Memos randomised controlled trial. *Br J Psychiatry*. 2012;200(5):412-418. doi:10.1192/bjp.bp.111.101394
171. Morgan AJ, Jorm AF, Mackinnon AJ. Self-Help for Depression via E-mail: A Randomised Controlled Trial of Effects on Depression and Self-Help Behaviour. *PLoS One*. 2013;8(6):1-10. doi:10.1371/journal.pone.0066537
172. Moritz S, Quan H, Rickhi B, et al. A home study-based spirituality education program decreases emotional distress and increases quality of life - A randomized, controlled trial. *Altern Ther Health Med*. 2006.
173. Moritz S, Schilling L, Hauschildt M, Schröder J, Treszl A. A randomized controlled trial of internet-based therapy in depression. *Behav Res Ther*. 2012;50(7-8):513-521. doi:10.1016/j.brat.2012.04.006
174. Morris RR, Schueller SM, Picard RW. Efficacy of a web-based, crowdsourced peer-to-peer cognitive reappraisal platform for depression: Randomized controlled trial. *J Med Internet Res*. 2015. doi:10.2196/jmir.4167
175. Mullin A, Dear BF, Karin E, et al. The UniWellbeing course: A randomised controlled trial of a transdiagnostic internet-delivered cognitive behavioural therapy (CBT) programme for university students with symptoms of anxiety and depression. *Internet Interv*. 2015;2(2):128-136. doi:10.1016/j.invent.2015.02.002
176. Nelson CB, Abraham KM, Walters H, Pfeiffer PN, Valenstein M. Integration of peer support and computer-based CBT for veterans with depression. *Comput Human Behav*. 2014;31(1):57-64. doi:10.1016/j.chb.2013.10.012
177. Newby JM, Mackenzie A, Williams AD, et al. Internet cognitive behavioural therapy for mixed anxiety and depression: A randomized controlled trial and evidence of effectiveness in primary care. *Psychol Med*. 2013;43(12):2635-2648. doi:10.1017/S0033291713000111
178. Newby JM, Lang T, Werner-Seidler A, Holmes E, Moulds ML. Alleviating distressing intrusive memories in depression: A comparison between computerised cognitive bias modification and cognitive behavioural education. *Behav Res Ther*. 2014;56(1):60-67. doi:10.1016/j.brat.2014.03.001
179. Newby JM, Mewton L, Andrews G. Transdiagnostic versus disorder-specific internet-delivered cognitive behaviour therapy for anxiety and depression in primary care. *J Anxiety Disord*. 2017;46:25-34. doi:10.1016/j.janxdis.2016.06.002
180. Newby JM, Mewton L, Williams AD, Andrews G. Effectiveness of transdiagnostic internet cognitive behavioural treatment for mixed anxiety and depression in primary care. *J Affect Disord*. 2014;165(May):45-52. doi:10.1016/j.jad.2014.04.037
181. Newby J, Robins L, Wilhelm K, et al. Web-based cognitive behavior therapy for depression in people with diabetes mellitus:A randomized controlled trial. *J Med Internet Res*. 2017;19(5). doi:10.2196/jmir.7274
182. Nicky M. Computerised CBT self-help for depression in higher education: Reflections on a pilot. *Couns Psychother Res*. 2009;9(4):280-286. doi:10.1080/14733140902993343
183. Nobis S, Lehr D, Ebert DD, et al. Efficacy of a web-based intervention with mobile phone support in treating depressive symptoms in adults with type 1 and type 2

diabetes: A randomized controlled trial. *Diabetes Care*. 2015;38(5):776-783. doi:10.2337/dc14-1728
184. Nørregaard LB, Løventoft PK, Frøkjær E, et al. Patient expectations and experiences from a clinical study in psychiatric care using a self-monitoring system. In: *Proceedings of the 8th Nordic Conference on Human-Computer Interaction: Fun, Fast, Foundational*. ; 2014:991-994. doi:10.1145/2639189.2670258
185. O' Leary K, Dockray S. The Effects of Two Novel Gratitude and Mindfulness Interventions on Well-Being. *J Altern Complement Med*. 2015;21(4):243-245. doi:10.1089/acm.2014.0119
186. Paredes P, Giald-Bachrach R, Czerwinski M, Roseway A, Rowan K, Hernandez J. PopTherapy: Coping with Stress through Pop-Culture. 2014. doi:10.4108/icst.pervasivehealth.2014.255070
187. Patten SB. Prevention of Depressive Symptoms Through the Use of Distance Technologies. *Psychiatr Serv*. 2003;54(3):396-398. doi:10.1176/appi.ps.54.3.396
188. Perini S, Titov N, Andrews G. Clinician-assisted Internet-based treatment is effective for depression: Randomized controlled trial. *Aust N Z J Psychiatry*. 2009;43:571-578.
189. Perini S, Titov N, Andrews G. The Climate Sadness program: an open trial of Internet-based treatment for depression. *E-Journal Appl Psychol*. 2013;4(2):18-24. doi:10.7790/ejap.v4i2.135
190. Peters KD, Constans JI, Mathews A. Experimental modification of attribution processes. *J Abnorm Psychol*. 2011;120(1):168-173. doi:10.1037/a0021899
191. Pfeiffer PN, Henry J, Ganoczy D, Piette JD. Pilot study of psychotherapeutic text messaging for depression. *J Telemed Telecare*. 2017;23(7):665-672. doi:10.1177/1357633X16659955
192. Phillips R, Schneider J, Molosankwe I, et al. Randomized controlled trial of computerized cognitive behavioural therapy for depressive symptoms: Effectiveness and costs of a workplace intervention. *Psychol Med*. 2014;44(4):741-752. doi:10.1017/S0033291713001323
193. Pictet A, Jermann F, Ceschi G. When less could be more: Investigating the effects of a brief internet-based imagery cognitive bias modification intervention in depression. *Behav Res Ther*. 2016;84(August):45-51. doi:10.1016/j.brat.2016.07.008
194. Piette JD, Aikens JE, Trivedi R, et al. Depression self-management assistance using automated telephonic assessments and social support. *Am J Manag Care*. 2013;19(11):892-900. http://www.ncbi.nlm.nih.gov/pubmed/24511987.
195. Pittaway PGCE S, Arowobusoye MFPH N, Milne RB, et al. Comparative, clinical feasibility study of three tools for delivery of cognitive behavioural therapy for mild to moderate depression and anxiety provided on a self-help basis. *Ment Health Fam Med*. 2009;6(September 2009):145-154. https://www.ncbi.nlm.nih.gov/pmc/articles/PMC2838647/pdf/MHFM-06-145.pdf.
196. Pots WTM, Fledderus M, Meulenbeek PAM, ten Klooster PM, Schreurs KMG, Bohlmeijer ET. Acceptance and commitment therapy as a web-based intervention for depressive symptoms: randomised controlled trial. *Br J Psychiatry*. 2016;208(1):69-77. doi:10.1192/bjp.bp.114.146068

197. Preschl B, Maercker A, Wagner B. The working alliance in a randomized controlled trial comparing online. *BMC Psychiatry*. 2011.
198. Proudfoot J, Swain S, Widmer S, et al. The development and beta-test of a computer-therapy program for anxiety and depression: Hurdles and lessons. *Comput Human Behav*. 2003;19(3):277-289. doi:10.1016/S0747-5632(02)00062-6
199. Proudfoot J, Clarke J, Birch M-R, et al. Impact of a mobile phone and web program on symptom and functional outcomes for people with mild-to-moderate depression, anxiety and stress: a randomised controlled trial. *BMC Psychiatry*. 2013;13(1):312. doi:10.1186/1471-244X-13-312
200. Proudfoot J, Goldberg D, Mann A, Everitt B, Marks I, Gray JA. Computerized, interactive, multimedia cognitive-behavioural program for anxiety and depression in general practice. *Psychol Med*. 2003;33(2):217-227. doi:10.1017/S0033291702007225
201. Proudfoot J, Ryden C, Everitt B, et al. Clinical efficacy of computerised cognitive-behavioural therapy for anxiety and depression in primary care: randomised controlled trial. *Br J Psychiatry*. 2004;185:46-54. http://www.ncbi.nlm.nih.gov/pubmed/15231555.
202. Proyer RT, Gander F, Wellenzohn S, Ruch W. Nine beautiful things: A self-administered online positive psychology intervention on the beauty in nature, arts, and behaviors increases happiness and ameliorates depressive symptoms. *Pers Individ Dif*. 2016;94:189-193. doi:10.1016/j.paid.2016.01.028
203. Proyer RT, Gander F, Wellenzohn S, Ruch W. Positive psychology interventions in people aged 50-79 years: Long-term effects of placebo-controlled online interventions on well-being and depression. *Aging Ment Heal*. 2014;18(8):997-1005. doi:10.1080/13607863.2014.899978
204. Purves DG, Bennett M, Wellman N. An Open Trial in the NHS of Blues Begone®: A New Home Based Computerized CBT Program. *Behav Cogn Psychother*. 2009;37(5):541-551. doi:10.1017/s1352465809990282
205. Räsänen P, Lappalainen P, Muotka J, Tolvanen A, Lappalainen R. An online guided ACT intervention for enhancing the psychological wellbeing of university students: A randomized controlled clinical trial. *Behav Res Ther*. 2016;78:30-42. doi:10.1016/j.brat.2016.01.001
206. Rebar AL, Boles C, Burton N, et al. Healthy mind, healthy body: A randomized trial testing the efficacy of a computer-tailored vs. interactive web-based intervention for increasing physical activity and reducing depressive symptoms. *Ment Health Phys Act*. 2016;11:29-37. doi:10.1016/j.mhpa.2016.08.001
207. Rice S, Gleeson J, Davey C, et al. Moderated online social therapy for depression relapse prevention in young people: pilot study of a 'next generation' online intervention. *Early Interv Psychiatry*. 2018;12(4):613-625. doi:10.1111/eip.12354
208. Richards D, Timulak L, O'Brien E, et al. A randomized controlled trial of an internet-delivered treatment: Its potential as a low-intensity community intervention for adults with symptoms of depression. *Behav Res Ther*. 2015;75(October):20-31. doi:10.1016/j.brat.2015.10.005
209. Richards D, Murphy T, Viganó N, et al. Acceptability, satisfaction and perceived efficacy of "Space from Depression" an internet-delivered treatment for depression. *Internet Interv*. 2016;5:12-22. doi:10.1016/j.invent.2016.06.007

210. Richards D, Timulak L, Hevey D. A comparison of two online cognitive-behavioural interventions for symptoms of depression in a student population: The role of therapist responsiveness. *Couns Psychother Res*. 2013;13(3):184-193. doi:10.1080/14733145.2012.733715
211. Robertson L, Smith M, Tannenbaum D. Case management and adherence to an online disease management system. *J Telemed Telecare*. 2005;11(2_suppl):73-75. doi:10.1258/135763305775124885
212. Robinson J, Hetrick S, Cox G, et al. Can an Internet-based intervention reduce suicidal ideation, depression and hopelessness among secondary school students: Results from a pilot study. *Early Interv Psychiatry*. 2016;10(1):28-35. doi:10.1111/eip.12137
213. Roepke AM, Jaffee SR, Riffle OM, McGonigal J, Broome R, Maxwell B. Randomized Controlled Trial of SuperBetter, a Smartphone-Based/Internet-Based Self-Help Tool to Reduce Depressive Symptoms. *Games Health J*. 2015;4(3):235-246. doi:10.1089/g4h.2014.0046
214. Rosso IM, Killgore WDS, Olson EA, et al. Internet-based cognitive behavior therapy for major depressive disorder: A randomized controlled trial. *Depress Anxiety*. 2017;34(3):236-245. doi:10.1002/da.22590
215. Ruwaard J, Schrieken B, Schrijver M, et al. Standardized Web-Based Cognitive Behavioural Therapy of Mild to Moderate Depression: A Randomized Controlled Trial with a Long-Term Follow-Up. *Cogn Behav Ther*. 2009;38(4):206-221. doi:10.1080/16506070802408086
216. Salisbury C, O'Cathain A, Edwards L, et al. Effectiveness of an integrated telehealth service for patients with depression: A pragmatic randomised controlled trial of a complex intervention. *The Lancet Psychiatry*. 2016;3(6):515-525. doi:10.1016/S2215-0366(16)00083-3
217. Salisbury C, Thomas C, O'Cathain A, et al. TElehealth in CHronic disease: Mixed-methods study to develop the TECH conceptual model for intervention design and evaluation. *BMJ Open*. 2015;5(2):1-12. doi:10.1136/bmjopen-2014-006448
218. Santucci LC, McHugh RK, Elkins RM, et al. Pilot implementation of computerized cognitive behavioral therapy in a university health setting. *Adm Policy Ment Heal Ment Heal Serv Res*. 2014;41(4):514-521. doi:10.1007/s10488-013-0488-2
219. Schneider J, Foroushani PS, Grime P, Thornicroft G. Acceptability of online self-help to people with depression: Users' views of moodgym versus informational websites. *J Med Internet Res*. 2014;16(3):1-13. doi:10.2196/jmir.2871
220. Schröder J, Brückner K, Fischer A, et al. Efficacy of a psychological online intervention for depression in people with epilepsy: A randomized controlled trial. *Epilepsia*. 2014;55(12):2069-2076. doi:10.1111/epi.12833
221. Schueller S, Mohr D. Initial Field Trial of a Coach-Supported Web-Based Depression Treatment. In: *Proceedings of the 9th International Conference on Pervasive Computing Technologies for Healthcare*. ICST; 2015. doi:10.4108/icst.pervasivehealth.2015.260115
222. Seligman MEP, Steen TA, Park N, Peterson C. Positive Psychology Progress: Empirical Validation of Interventions. *Am Psychol*. 2005;60(5):410-421. doi:10.1037/0003-066X.60.5.410

223. Seligman MEP, Schulman P, Tryon AM. Group prevention of depression and anxiety symptoms. *Behav Res Ther*. 2007;45(6):1111-1126. doi:10.1016/j.brat.2006.09.010
224. Sergeant S, Mongrain M. An online optimism intervention reduces depression in pessimistic individuals. *J Consult Clin Psychol*. 2014;82(2):263-274. doi:10.1037/a0035536
225. Sethi S. Treating youth depression and anxiety: A randomised controlled trial examining the efficacy of computerised versus face-to-face cognitive behaviour therapy. *Aust Psychol*. 2013;48(4):249-257. doi:10.1111/ap.12006
226. Shamekhi A, Bickmore T, Lestoquoy A, Gardiner P. Augmenting Group Medical Visits with Conversational Agents for Stress Management Behavior Change. 2017:55-67. doi:10.1007/978-3-319-55134-0_5
227. Shamekhi A, Bickmore T, Lestoquoy A, Negash L, Gardiner P. Blissful Agents: Adjuncts to Group Medical Visits for Chronic Pain and Depression. In: *Lecture Notes in Computer Science (Including Subseries Lecture Notes in Artificial Intelligence and Lecture Notes in Bioinformatics)*. ; 2016:433-437. doi:10.1007/978-3-319-47665-0_49
228. Shapira LB, Mongrain M. The benefits of self-compassion and optimism exercises for individuals vulnerable to depression. *J Posit Psychol*. 2010;5(5):377-389. doi:10.1080/17439760.2010.516763
229. Sharry J, Davidson R, McLoughlin O, Doherty G. A service-based evaluation of a therapist-supported online cognitive behavioral therapy program for depression. *J Med Internet Res*. 2013;15(6):1-14. doi:10.2196/jmir.2248
230. Skolarus LE, Piette JD, Pfeiffer PN, et al. Interactive Voice Response—An Innovative Approach to Post-Stroke Depression Self-Management Support. *Transl Stroke Res*. 2017;8(1):77-82. doi:10.1007/s12975-016-0481-7
231. Spates CR, Kalata AH, Ozeki S, Stanton CE, Peters S. Initial Open Trial of a Computerized Behavioral Activation Treatment for Depression. *Behav Modif*. 2013;37(3):259-297. doi:10.1177/0145445512455051
232. SPEK V, NYKLÍČEK I, SMITS N, et al. Internet-based cognitive behavioural therapy for subthreshold depression in people over 50 years old: a randomized controlled clinical trial. *Psychol Med*. 2007;37(12):1797-1806. doi:10.1017/S0033291707000542
233. Staples LG, Fogliati VJ, Dear BF, Nielssen O, Titov N. Internet-delivered treatment for older adults with anxiety and depression: implementation of the Wellbeing Plus Course in routine clinical care and comparison with research trial outcomes. *BJPsych Open*. 2016;2(5):307-313. doi:10.1192/bjpo.bp.116.003400
234. Ström M, Uckelstam C-J, Andersson G, Hassmén P, Umefjord G, Carlbring P. Internet-delivered therapist-guided physical activity for mild to moderate depression: a randomized controlled trial. *PeerJ*. 2013;1(2013):e178. doi:10.7717/peerj.178
235. Thompson NJ, Walker ER, Obolensky N, et al. Distance delivery of mindfulness-based cognitive therapy for depression: Project UPLIFT. *Epilepsy Behav*. 2010;19(3):247-254. doi:10.1016/j.yebeh.2010.07.031
236. Tiburcio M, Lara MA, Aguilar Abrego A, Fernández M, Martínez Vélez N, Sánchez A. Web-Based Intervention to Reduce Substance Abuse and Depressive

Symptoms in Mexico: Development and Usability Test. *JMIR Ment Heal*. 2016;3(3):e47. doi:10.2196/mental.6001

237. Titov N, Dear BF, Staples LG, et al. Disorder-specific versus transdiagnostic and clinician-guided versus self-guided treatment for major depressive disorder and comorbid anxiety disorders: A randomized controlled trial. *J Anxiety Disord*. 2015;35:88-102. doi:10.1016/j.janxdis.2015.08.002
238. Titov N, Andrews G, Davies M, McIntyre K, Robinson E, Solley K. Internet Treatment for Depression: A Randomized Controlled Trial Comparing Clinician vs. Technician Assistance. *PLoS One*. 2013;5(6):1-9. doi:10.1371/journal.pone.0010939
239. Titov N, Dear BF, Johnston L, et al. Improving adherence and clinical outcomes in self-guided internet treatment for anxiety and depression: a 12-month follow-up of a randomised controlled trial. *PLoS One*. 2014;9(2):e89591. doi:10.1371/journal.pone.0089591
240. Titov N, Dear BF, Johnston L, et al. Improving Adherence and Clinical Outcomes in Self-Guided Internet Treatment for Anxiety and Depression: Randomised Controlled Trial. *PLoS One*. 2013;8(7). doi:10.1371/journal.pone.0062873
241. Titov N, Dear BF, Schwencke G, et al. Transdiagnostic internet treatment for anxiety and depression: A randomised controlled trial. *Behav Res Ther*. 2011;49(8):441-452. doi:10.1016/j.brat.2011.03.007
242. Titov N, Fogliati VJ, Staples LG, et al. Treating anxiety and depression in older adults: randomised controlled trial comparing guided V. self-guided internet-delivered cognitive–behavioural therapy. *BJPsych Open*. 2016;2(1):50-58. doi:10.1192/bjpo.bp.115.002139
243. Titov, N, Dear, et al. Clinical and cost-effectiveness of therapist-guided internet-delivered cognitive behavior therapy for older adults with symptoms of depression: a randomized controlled trial (Provisional abstract). *Behav Ther*. 2014;46(2):206-217.
244. Topolovec-Vranic J, Cullen N, Michalak A, et al. Evaluation of an online cognitive behavioural therapy program by patients with traumatic brain injury and depression. *Brain Inj*. 2010;24(5):762-772. doi:10.3109/02699051003709599
245. Torkan H, Blackwell SE, Holmes EA, et al. Positive imagery cognitive bias modification in treatment-seeking patients with major depression in Iran: A pilot study. *Cognit Ther Res*. 2014;38(2):132-145. doi:10.1007/s10608-014-9598-8
246. Twomey C, O'Reilly G, Byrne M, et al. A randomized controlled trial of the computerized CBT programme, MoodGYM, for public mental health service users waiting for interventions. *Br J Clin Psychol*. 2014;53(4):433-450. doi:10.1111/bjc.12055
247. Ünlü Ince B, Cuijpers P, Van'T Hof E, Van Ballegooijen W, Christensen H, Riper H. Internet-based, culturally sensitive, problem-solving therapy for Turkish migrants with depression: Randomized controlled trial. *J Med Internet Res*. 2013;15(10):1-16. doi:10.2196/jmir.2853
248. Van Bastelaar KMP, Pouwer F, Cuijpers P, Riper H, Snoek FJ. Web-based depression treatment for type 1 and type 2 diabetic patients: A randomized, controlled trial. *Diabetes Care*. 2011;34(2):320-325. doi:10.2337/dc10-1248
249. Van Den Berg S, Shapiro DA, Bickerstaffe D, Cavanagh K. Computerized cognitive-behaviour therapy for anxiety and depression: A practical solution to

the shortage of trained therapists. *J Psychiatr Ment Health Nurs*. 2004;11(5):508-513. doi:10.1111/j.1365-2850.2004.00745.x

250. Van Der Zanden R, Kramer J, Gerrits R, Cuijpers P. Effectiveness of an online group course for depression in adolescents and young adults: A randomized trial. *J Med Internet Res*. 2012;14(3):1-15. doi:10.2196/jmir.2033
251. Van Dongen AJCM, Nelen WLDM, Inthout J, Kremer JAM, Verhaak CM. e-Therapy to reduce emotional distress in women undergoing assisted reproductive technology (ART): A feasibility randomized controlled trial. *Hum Reprod*. 2016;31(5):1046-1057. doi:10.1093/humrep/dew040
252. Van Spijker BAJ, Van Straten A, Kerkhof AJFM. Effectiveness of online self-help for suicidal thoughts: Results of a randomised controlled trial. *PLoS One*. 2014;9(2). doi:10.1371/journal.pone.0090118
253. Van Straten A, Cuijpers P, Smits N. Effectiveness of a web-based self-help intervention for symptoms of depression, anxiety, and stress: Randomized controlled trial. *J Med Internet Res*. 2008;10(1). doi:10.2196/jmir.954
254. Van Voorhees BW, Ellis JM, Gollan JK, et al. Development and process evaluation of a primary care internet-based intervention to prevent depression in emerging adults. *Prim Care Companion J Clin Psychiatry*. 2007;9(5):346-355. http://www.ncbi.nlm.nih.gov/pubmed/17998953%0Ahttp://www.pubmedcentral.nih.gov/articlerender.fcgi?artid=PMC2040278.
255. Vernmark K, Lenndin J, Bjärehed J, et al. Internet administered guided self-help versus individualized e-mail therapy: A randomized trial of two versions of CBT for major depression. *Behav Res Ther*. 2010;48(5):368-376. doi:10.1016/j.brat.2010.01.005
256. Wagner B, Horn AB, Maercker A. Internet-based versus face-to-face cognitive-behavioral intervention for depression: A randomized controlled non-inferiority trial. *J Affect Disord*. 2014;152-154(1):113-121. doi:10.1016/j.jad.2013.06.032
257. Wahle F, Kowatsch T, Fleisch E, Rufer M, Weidt S. Mobile Sensing and Support for People With Depression: A Pilot Trial in the Wild. *JMIR mHealth uHealth*. 2016;4(3):e111. doi:10.2196/mhealth.5960
258. Warmerdam L, Van Straten A, Twisk J, Riper H, Cuijpers P. Internet-based treatment for adults with depressive symptoms: Randomized controlled trial. *J Med Internet Res*. 2008;10(4):1-12. doi:10.2196/jmir.1094
259. Watts S, Mackenzie A, Thomas C, et al. CBT for depression: A pilot RCT comparing mobile phone vs. computer. *BMC Psychiatry*. 2013. doi:10.1186/1471-244X-13-49
260. Wellenzohn S, Proyer RT, Ruch W. Humor-based online positive psychology interventions: A randomized placebo-controlled long-term trial. *J Posit Psychol*. 2016;11(6):584-594. doi:10.1080/17439760.2015.1137624
261. Whiteside U. Online Cognitive Behavioral Therapy for Depressed Primary Care Patients: A Pilot Feasibility Project. *Perm J*. 2014;18(2):21-27. doi:10.7812/tpp/13-155
262. Whitfield G, Hinshelwood R, Pashely A, Campsie L, Williams C. The Impact of a Novel Computerized CBT CD Rom (Overcoming Depression) Offered to Patients Referred to Clinical Psychology. *Behav Cogn Psychother*. 2006;34(1):1-11. doi:10.1017/s135246580500250x

263. Williams AD, Andrews G. The Effectiveness of Internet Cognitive Behavioural Therapy (iCBT) for Depression in Primary Care: A Quality Assurance Study. *PLoS One*. 2013;8(2). doi:10.1371/journal.pone.0057447
264. Williams AD, Blackwell SE, Mackenzie A, Holmes EA, Andrews G. Combining imagination and reason in the treatment of depression: A randomized controlled trial of internet-based cognitive-bias modification and internet-CBT for depression. *J Consult Clin Psychol*. 2013;81(5):793-799. doi:10.1037/a0033247
265. Williams AD, O'Moore K, Blackwell SE, Smith J, Holmes EA, Andrews G. Positive imagery cognitive bias modification (CBM) and internet-based cognitive behavioral therapy (iCBT): A randomized controlled trial. *J Affect Disord*. 2015;178:131-141. doi:10.1016/j.jad.2015.02.026
266. Williams AD, Thompson J, Andrews G. The impact of psychological distress tolerance in the treatment ofdepression. *Behav Res Ther*. 2013;51(8):469-475. doi:10.1016/j.brat.2013.05.005
267. Wright AS, Salmon P, Beck AT, Kuykendall J, Goldsmith LJ, Zickel MB. Development and Initial Testing of a Multimedia Program for Computer-assisted Cognitive Therapy. *Am J Psychother*. 2002;56(1):76-86.
268. Wright JH, Wright AS, Albano AM, et al. Computer-assisted cognitive therapy for depression: Maintaining efficacy while reducing therapist time. *Am J Psychiatry*. 2005;162(6):1158-1164. doi:10.1176/appi.ajp.162.6.1158
269. Wu S, Vidyanti I, Liu P, et al. Patient-centered technological assessment and monitoring of depression for low-income patients. *J Ambul Care Manage*. 2014. doi:10.1097/JAC.0000000000000027
270. Yiend J, Lee JS, Tekes S, et al. Modifying interpretation in a clinically depressed sample using "cognitive bias modification-errors": A double blind randomised controlled trial. *Cognit Ther Res*. 2014;38(2):146-159. doi:10.1007/s10608-013-9571-y
