# Supplementary figures and images for: Technological State of the Art of Electronic Mental Health Interventions for Major Depressive Disorder: Systematic Literature Review
Source: J Med Internet Res. 2020 Jan 20;22(1):e12599. doi: 10.2196/12599 (PMC6997926; doi:10.2196/12599)

# Software Systems for Depression

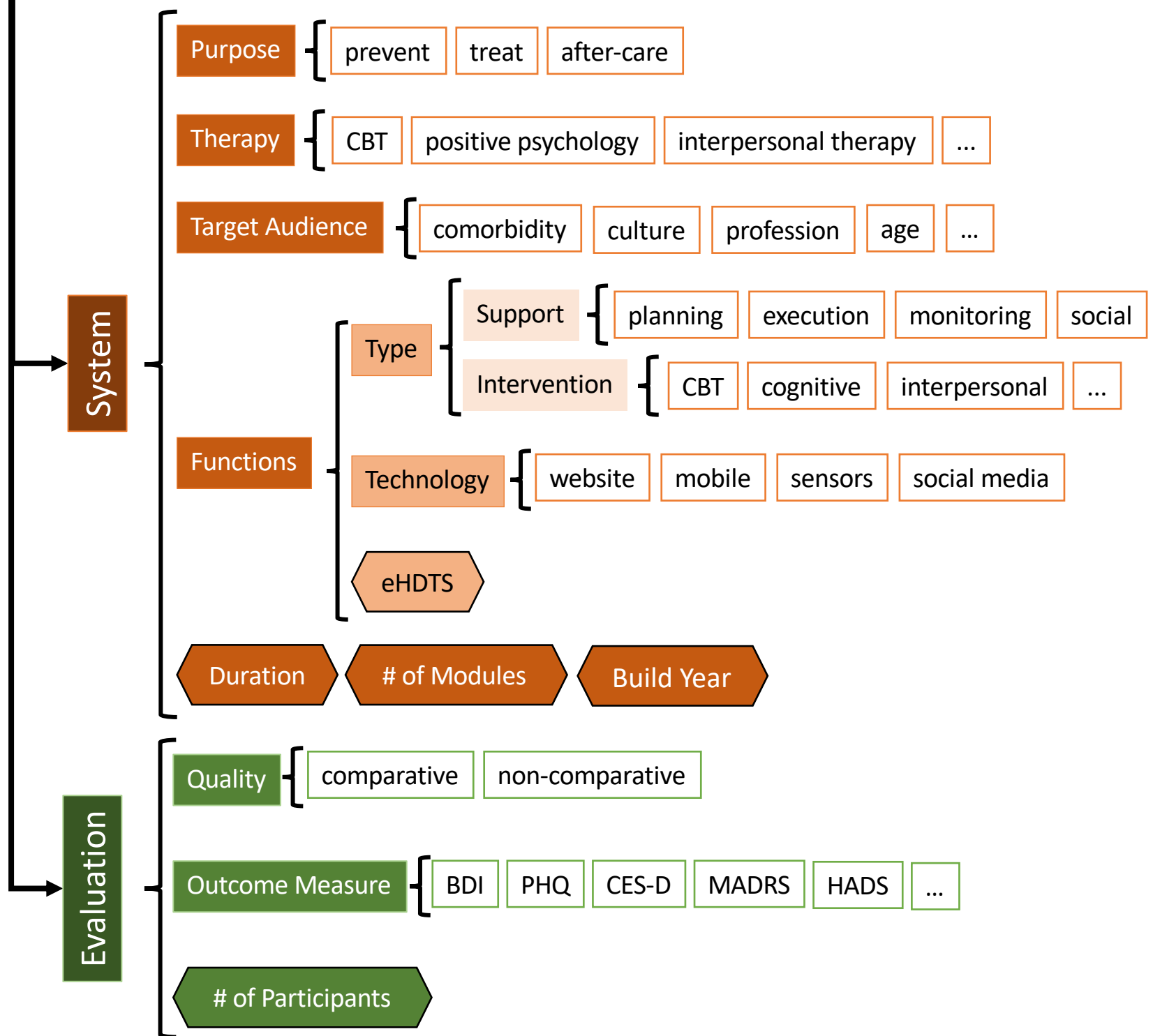

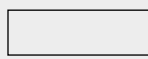 qualitative attributes

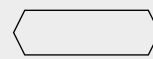 quantitative attributes

Supplement: Multimedia Appendix 3 [file jmir_v22i1e12599_app3.pdf]

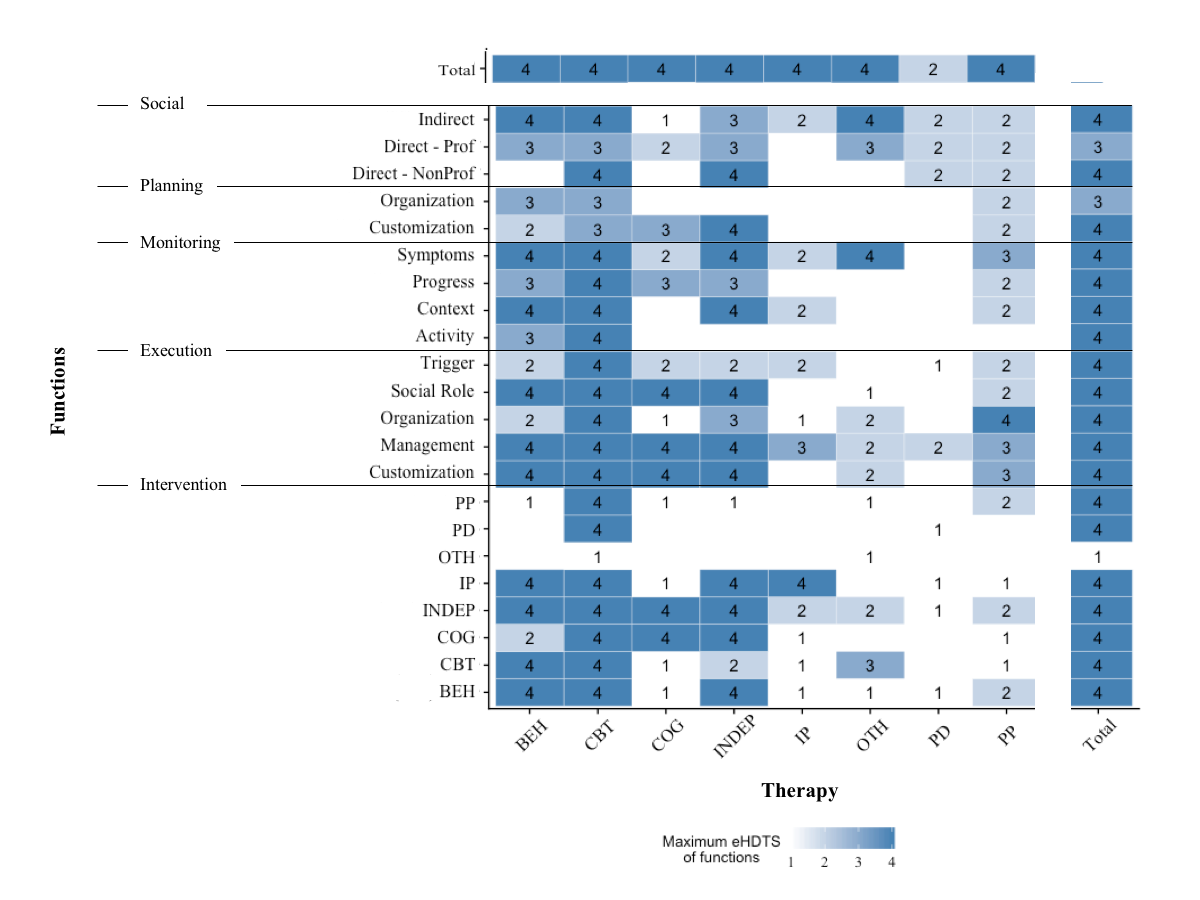

Supplement: Multimedia Appendix 10 [file jmir_v22i1e12599_app10.png]
