# Supplementary material for: Technological State of the Art of Electronic Mental Health Interventions for Major Depressive Disorder: Systematic Literature Review
Source: J Med Internet Res. 2020 Jan 20;22(1):e12599. doi: 10.2196/12599 (PMC6997926; doi:10.2196/12599)
Supplement: Multimedia Appendix 4 [file jmir_v22i1e12599_app4.pdf]

L0	L1	L2
User preferences (intervention/system)	Customization	Execution
Tunneling, Intervention reasoning, Risk management, Troubleshooting	Management	Execution
Scheduling, Goal setting, Self-screening	Organization	Execution
Rewarding, Encouraging, Empathy, Similarity, Authority, Consideration	Social Role	Execution
Signal, Spark, Facilitator	Trigger	Execution
Automatic/Manual activity monitoring	Activity	Monitoring
Automatic/Manual context monitoring	Context	Monitoring
Automatic/Manual progress monitoring	Progress	Monitoring
Automatic/Manual symptom monitoring	Symptoms	Monitoring
User preferences (intervention/system)	Customization	Planning
Goal setting, Tutorial	Organization	Planning
Peer support, Administrative support, Lay-person support	Direct: NonProfessional	Social
Nurse support, Professional support, Therapist support	Direct: Professional	Social
Social learning/comparison/facilitation, Recognition, Cooperation	Indirect	Social
