# Supplementary material for: Technological State of the Art of Electronic Mental Health Interventions for Major Depressive Disorder: Systematic Literature Review
Source: J Med Internet Res. 2020 Jan 20;22(1):e12599. doi: 10.2196/12599 (PMC6997926; doi:10.2196/12599)
Supplement: Multimedia Appendix 5 [file jmir_v22i1e12599_app5.pdf]

L0 Example	Therapeutic Framework	L1
Mood monitoring training	Cognitive Behavioral Therapy	Cognitive-Behavioral
Increase self-esteem and acceptance	Acceptance & Commitment Therapy	Cognitive-Behavioral
Learning to be mindful of moods and emotions	Mindfulness(-Based Stress Reduction)	Cognitive-Behavioral
Behavior pattern identification	Behavioral Activation	Behavioral
Breaking a task down into manageable parts	Problem Solving	Behavioral
Graded exposure	Exposure Therapy	Behavioral
-	Social Cognitive Theory	Behavioral
Increase physical activity	Physical Activity	Behavioral
Notice the effects of automatic thoughts	Cognitive Therapy	Cognitive
Concreteness training	Cognitive Bias Modification Training	Cognitive
Cognitive control training	Cognitive Control Training	Cognitive
Learning to recognize threat-monitoring	Meta-Cognitive Therapy	Cognitive
-	Cognitive Remediation	Cognitive
Childhood and early schemata reprocessing	Schema Therapy	Cognitive
Assertiveness and communication skills	Interpersonal Therapy	Interpersonal
Reflecting on past successes	Positive Psychology	Positive Psychology
Breaking unhelpful unconscious patterns	Psychodynamic	Psychodynamic
Building the motivation for change	Motivational Interviewing	Independent
Psychoeducation on depression	Bibliotherapy	Independent
-	Symptom Monitoring	Independent
Expressive Writing	Expressive Writing	Independent
Preventing relapses	Transdiagnostic	Independent
Mind/Body Programming	Hypnosis	Other
Life Review	Life Review	Other
Spiritual and religious resources	Spirituality	Other
