# Supplementary material for: Technological State of the Art of Electronic Mental Health Interventions for Major Depressive Disorder: Systematic Literature Review
Source: J Med Internet Res. 2020 Jan 20;22(1):e12599. doi: 10.2196/12599 (PMC6997926; doi:10.2196/12599)
Supplement: Multimedia Appendix 6 [file jmir_v22i1e12599_app6.pdf]

Website of the ehealth4mdd Database

e-Health degree of technological sophistication scales

This table lists the five different eHDTS scales, defines each level, provides an example function for each level and each scale as well as the inter-rater agreement that was obtained when calculating Cohen's kappa between two raters that rated a sample of approximately 25 function descriptions (similar to the examples) using the scale level definitions.

Scale Name	Abbreviation	Level 0	Level 1	Level 2	Level 3	Level 4
Intervention	eHDTS_i	functionality is provided by a human (e.g. face-to-face, by email or telephone)	functionality is not interactive but purely informational (e.g. downloadable pdf, information website)	functionality is provided in an interactive manner (e.g. the user can fill in information on a website) but the information is not processed by the system in any way	functionality is provided in an interactive manner and input from the user is responded to but without processing content of input (e.g. more information is presented after the user has typed a certain the number of characters)	functionality is provided in an interactive manner; content of user input is processed and feedback is provided
Example	*eHDTS_i*	*[Function: Activity planning] [Platform: FACE-TO-FACE] [Description: create an activity plan] [Implementation Details:]*	*[Function: Activity planning][Platform: ONLINE] [Description: Instructions about controlling physical symptoms using strategies such as controlled breathing and scheduling pleasant activities.] [Implementation Details:]*	*[Function: Activity planning] [Platform: ONLINE] [Description: schedule positive activities] [Implementation Details: Each lesson was paired with an interactive tool to provide participants with opportunities to apply the treatment concepts discussed in the lesson. Examples include an activity calendar with which participants could monitor and schedule their activities. These tools were designed to be completed in just a few minutes.]*	*[Function: Activity planning][Platform: APP] [Description: Daybuilder includes a calendar, with one novel facility called "event types". Event types are used for modeling events that require some extra actions. For example, when going to a wedding one has to be nicely dressed and maybe buy a gift. The Daybuilder calendar lets the user create event types and use them to be reminded of the extra actions required for the event.] [Implementation Details:]*	*[Function: Activity planning] [Platform: ONLINE] [Description: a website tutorial guides users through identification of eight to 12 pleasant activities that matter most to their mood (Figure 2). The website then assists with creating a personalized self-contract to make small but consistent increases in the frequency of these activities. Users are prompted to return to the website every few days to record total daily activities and a daily mood rating (on a 7-point Likert scale) for each intervening day.] [Implementation Details: Website algorithms process these tracking data to generate personalized feedback regarding the*

						association (or lack of) between each user's daily mood and his or her pleasant activity level. The website identifies those activities that users report occurring infrequently or not at all or those that are not associated with increased mood, and suggests that these might be replaced with other, more mood-lifting activities.]
Planning Support	eHDTS_p	functionality provided by a human either through the system or entirely externally	functionality provided in a non-interactive/informational manner (e.g. the user is only informed of possible paths through the intervention but the system does not adapt in any manner)	functionality is provided in an interactive manner but no support, this includes also user-driven tailoring if all users are provided with the same options to choose from (e.g. the user can set certain parameters to personalize the system)	functionality is provided with tailored support, tailoring on the basis of interactions with the system	functionality is provided with personalized, context-aware support (e.g. by integrating with Google calendar for scheduling)
Example	*eHDTS_p*	*[Function: Goal setting] [Platform: TELEPHONE] [Description: After an initial assessment and goal-setting telephone call] [Implementation Details:]*	*[Function: Goal setting] [Platform: EMAIL] [Description: Introduction, goals] [Implementation Details: During each online session, participants were sent approximately 20 to 30 PowerPoint slides, including general information on a particular topic, an overview of helpful skills related to the issue being covered, and homework sheets in Farsi (participants' first language).]*	*[Function: Goal setting] [Platform: ONLINE] [Description: SB users interacted with a game-like platform and were invited to describe a goal (an epic win; here, overcoming depression)] [Implementation Details:]*	*[Function: User preferences (intervention)] [Platform: COMPUTER] [Description: Item content covered seven categories: academic, family, mood and health, relationships, social activities, hobbies and work. In order to maximise the personal relevance of the intervention all participants were asked to indicate at screening which one of these categories was least relevant to them. For those subsequently assigned to the active condition, randomisation included automatic allocation to a personalised version of the intervention programme, that omitted all items in a participant's least*	*[Function: User preference (system)] [Platform: INTERACTIVE VOICE RESPONSE] [Description: Outbound automated calls are scheduled based on a dynamic protocol of type of call (screening or monitoring), calendar dates, clinical events, call history, and patient preferences (such as call time), using data and the DCAT algorithms in the DMR. Patients can opt for password-protected access to enhance*

					relevant category.] [Implementation Details:]	*privacy and can use the system to reschedule the call or request human follow-up.] [Implementation Details:]*
Execution Support	eHDTS_e	functionality provided by a human either through the system or externally	functionality provided in an informational manner or functionality is provided in a rule-based fashion (if-then), condition is user-independent (e.g. if Monday, release module)	functionality provided in a rule-based fashion (if-then), consequence is dependent on user interaction data (e.g. if user has not interacted with system for 7 days, send reminder)	functionality provided in a rule-based fashion (if-then), consequence is dependent on a static user model (e.g. if user is severely depressed, release new modules twice a week instead of once for mildly and moderately depressed users)	functionality is provided in a rule-based fashion (if-then), but consequence is dependent on a dynamically changing user model (e.g. system checks whether user has acquired new skills and if so those modules that require the application of these skills become accessible)
Example	*eHDTS_e*	*[Function: Tunneling] [Platform: ONLINE] [Description: Participants were given gradual access to the self-help modules. The therapists gave feedback on the clients' experiences and administered the gradual access to the modules.] [Implementation Details:]*	*[Function: Tunneling] [Platform: ONLINE] [Description: The participant logged into the program and was instructed to finish the lessons in a predetermined order.] [Implementation Details:]*	*[Function: Tunneling] [Platform: ONLINE] [Description: in the latest version of the MoodGYM program (Mark III) core assessments are compulsory not allowing the user to skip or alternate between the different modules.] [Implementation Details:]*	*[Function: Tunneling][Platform: ONLINE] [Description: The 20-item CES-D was the first level assessment in HIV TIDES. CES-D scores were used for the intervention pathways because the instrument is multi-factorial thus facilitating tailoring of specific self-care interventions. The cut-off points of 8 and 16 (total range from 0 to 60) were used to divide system users into three major groups which determined the tailoring path the users would experience.] [Implementation Details:]*	*[Function: Tunneling] [Platform: COMPUTER] [Description: Session 5 included a comprehensive assessment to evaluate whether participants had acquired sufficient knowledge to progress to the next five sessions or if remediation was warranted. The program utilized an algorithm that branched to earlier sessions when remedial learning objectives needed to be met based on scores obtained in Session 5.] [Implementation Details: Mini-lessons were prioritized based on the comprehensive assessment of participant needs during Session 5. Those content areas showing greatest need based on item score sampling*

						across all mini-lesson options (16 in total) were given highest priority and presented earlier and those with lower scores presented later.]
Monitoring Support	eHDTS_m	data collected but not used in intervention (e.g. data is collected for study purposes)	data collected and forwarded to human third party for use in intervention (e.g. a therapist receives data and makes choices accordingly)	data collected and presented to the user for self-monitoring (data is not interpreted by the system)	data collected and used to provide feedback to users on this specific data	data collected, analyzed, and used by system in the intervention (e.g. for personalization, intervention improvement, making prognoses)
Example	*eHDTS_m*	*[Function: Manual monitoring of symptoms] [Platform: SMS] [Description: The following mood monitoring message was sent once daily at random times: What is your mood right now on a scale of 1 to 10?] [Implementation Details:]*	*[Function: Manual monitoring of symptoms][Platform: ONLINE] [Description: Prior to each lesson, the patient completed the Kessler Psychological Distress Scale(K10) (Kessler et al.,2003) as a means of tracking patient progress during the iCBT course.] [Implementation Details: Clinicians were automatically notified if their patient's K10 score increased by half a standard deviation or more.]*	*[Function: Manual monitoring of symptoms] [Platform: ONLINE] [Description: Activity report for self-monitoring, which provides feedback to users, showing that their mood is related to the activities performed, and the benefits of being active.] [Implementation Details:]*	*[Function: Manual monitoring of symptoms][Platform: ONLINE] [Description: rating mood by selecting the extent to which each of 20 interactive mood-adjective playing cards describes current mood. The rating is based on the extent that users currently experience the 20 (positive and negative) emotions (e.g. proud, nervous, determined). Cards appear one at a time and the user selects either the flip or rotate button until their selection (very slightly or not at all, a little, quite a bit, extremely) is highlighted. They then click their selection and the next card appears. Test completion takes approximately 5 min and generates a daily score to enable mood tracking over time. Historical scores can be retrieved as a graph to which the user can add annotations. Users receive two forms of feedback on mood: automated feedback from the website, which comprises a short, two-paragraph supportive summation based on comparisons between the most*	*[Function: Manual monitoring of symptoms] [Platform: COMPUTER] [Description: The aim of this application is to detect symptoms of anxiety and depression in users. It includes a decision algorithm that allows Butler to react in real time to the user's clinical needs. To do this, an evaluation using widely used and validated psychological scales is performed when the user accesses the system. If the clinical status of the person is within the normal range, Butler offers its full potential. In the case that any change in mood (anxiety or depression) is detected, the system performs a more detailed exploration; depending on the result, it offers the most appropriate options for the user's*

					recent and previous scores (e.g. things aren't as good as they looked the last time you took the test and got 35%).] [Implementation Details:]	*emotional state and sends the appropriate warning to the professional user platform. This application also summarizes the information so the professional can process it efficiently through period analyses, data tables, bar graphics, and more.] [Implementation Details:]*
Social Support	eHDTS_s	human support is part of intervention but not integrated into system (e.g. system is an adjunct to face-to-face therapy) or the support is initiated by the support-provider at pre-specified times (e.g. therapists always sends feedback email to user after homework submission)	system provides support in a non-interactive/informational manner (e.g. it provides information on how to get in touch with human support or it includes videos of role models or summary statistics of how other users of the same system are doing)	system has interactive, dynamic, integrated social support (e.g. integrated chat functionality, integrated video conferencing, a peer support forum)	system is proactive about contacting human support on the basis of user activity in the system (e.g. it automatically contact support in case of risk detection, or it suggest to the user to cooperate with others on the basis of detected low adherence)	system intelligently supports the provision of human support by going beyond the specific user (e.g. by integrating with other social platforms and connecting a user with other people)
Example	*eHDTS_s*	*[Function: Nurse support] [Platform: TELEPHONE] [Description: Both groups continued nurse-supervised treatment for 12 weeks, which involved regular phone calls checking for problems] [Implementation Details:]*	*[Function: Social learning][Platform: ONLINE][Description: The Course comprises material both in didactic form, that is, text based instructions and information, and case-enhanced learning examples. Case-enhanced learning is informed by principles of Social Cognitive Theory (SCT) [33] and uses educational stories that identify a problem and a solution which an example (i.e., a case) resolves for the learner.] [Implementation Details:]*	*[Function: Social facilitation] [Platform: ONLINE] [Description: As a first step, the user can see anonymous indications of other people in the system. The intention is to reassure users that they are not alone in experiencing difficulties and that many other people have experienced similar problems and overcome them.] [Implementation Details: Users can respond to content by indicating that they "like" it, and can see how many other people liked it, helping to reduce the*	*[Function: Lay person support] [Platform: INTERACTIVE VOICE RESPONSE/EMAIL] [Description: based on subject's weekly IVR assessments, the CarePartner received structured emails or IVR calls with feedback about the subject's status with suggestions for how the CarePartner could support depressive symptom self-management. CarePartners were notified immediately via an IVR call if the subject reported (1) suicidal thoughts or a suicidal plan, (2) rarely or never taking the depression medication as prescribed, and (3) side effects that are making the subject consider taking less medication. Structured fax alerts*	*[Function: Cooperation] [Platform: SOCIAL MEDIA] [Description: Respondents in our study were a mixture of other Panoply users and paid workers from MTurk. Responses on Panoply fall into three categories: support, debug, and reframe. Support responses offer emotional support and active listening. Debug responses help users identify and dispute cognitive distortions (bugs). Reframe responses offer alternative, more positive ways of thinking*

				sense of isolation.]	*were sent automatically to the subject's PCP if the subject reported (1) medication non-adherence or(2) PHQ-9 >15 twice in the last month] [Implementation Details:]*	*about the stressful situation. Respondents are not asked to use any one particular reappraisal strategy but instead are given a bulleted list of tactics to consider in case they need inspiration.] [Implementation Details: All of the aforementioned interactions are coordinated entirely through Panoply's automation. The user needs to only submit their post to start this sequence of crowd work.]*
