# Supplementary material for: Technological State of the Art of Electronic Mental Health Interventions for Major Depressive Disorder: Systematic Literature Review
Source: J Med Internet Res. 2020 Jan 20;22(1):e12599. doi: 10.2196/12599 (PMC6997926; doi:10.2196/12599)
Supplement: Multimedia Appendix 7 [file jmir_v22i1e12599_app7.pdf]

Table 1. Systems evaluated in comparative trials ranked by evidence base.

System Key	*Name of System*	*Number of Versions*	*Evaluation Quality*	*Number of Evaluations*	*Number of Participants Pre*	*Number of Participants Post*
13	MoodGYM	15	comparative	11	7294	2636
16	Sadness Program	13	comparative	9	2738	1495
32	Alles onder controle	8	comparative	9	1960	1498
10	The Wellbeing Course	18	comparative	9	1879	1771
15	Beating the Blues	5	comparative	9	1422	1036
24	Andersson Unnamed	9	comparative	7	1422	1214
2	Deprexis	3	comparative	6	1863	1377
20	Colour your life	4	comparative	6	1574	1189
36	CBM Blackwell	6	comparative	5	376	347
14	e-couch	4	comparative	3	2703	1256
40	Get.On Mood Enhancer	3	comparative	3	1072	906
51	Living to the full	2	comparative	3	851	676
11	Overcoming Depression on the Internet	4	comparative	3	714	161
3	SHADE	2	comparative	3	475	56
54	Good Life Compass	2	comparative	3	145	139
34	Mood Memos	1	comparative	2	3062	1143
1	Living Life to the Full	2	comparative	2	659	575
115	Staerkentraining	5	comparative	2	623	462
18	Smiling is Fun	5	comparative	2	356	294
82	Cukrowicz Unnamed	2	comparative	2	242	119
129	CBM Moebius	1	comparative	2	191	191
78	Ly Unnamed	2	comparative	2	174	163
37	CBM-errors	2	comparative	2	120	114

109	Joutsenniemi Unnamed	1	comparative	1	3274	1288
98	UTSMed	1	comparative	1	1236	1020
133	Wellenzohn Unnamed	5	comparative	1	1162	994
53	SHUTi	1	comparative	1	1149	581
132	Shapira Unnamed	2	comparative	1	1002	188
46	Patten Unnamed	1	comparative	1	786	NA
65	Internet CBT program; Useful mental health solutions series for business.	1	comparative	1	762	606
104	Bunge Unnamed	4	comparative	1	728	464
61	myCompass	1	comparative	1	720	519
92	Seligman PPI	1	comparative	1	577	411
116	MyPAA	1	comparative	1	514	97
117	Walk 2.0	1	comparative	1	514	97
108	MH-Guru	1	comparative	1	507	386
93	Sergeant PPI	1	comparative	1	466	166
76	Geisner Unnamed	1	comparative	1	349	311
7	INCPAD	1	comparative	1	309	246
30	MoodHacker	1	comparative	1	300	286
74	Psyfit	1	comparative	1	284	214
39	SuperBetter	2	comparative	1	283	74
63	Depression Free	1	comparative	1	239	193
119	van Spijker Unnamed	1	comparative	1	236	215
47	SUMMIT	2	comparative	1	236	205
23	Seligman Unnamed	1	comparative	1	227	212
87	Panoply	1	comparative	1	217	166
31	Mindful Mood Balance	1	comparative	1	200	153
83	Tame Your Gut	1	comparative	1	199	143

85	Wellness Workshop	1	comparative	1	191	190
27	Space from Depression	1	comparative	1	188	152
90	Spirituality teaching program	1	comparative	1	165	147
118	Shamekhi Unnamed	1	comparative	1	160	154
72	Recovery Road	3	comparative	1	140	93
21	Creating Opportunities for Personal Empowerment	2	comparative	1	121	93
33	Digicoach	1	comparative	1	109	80
124	Learning Mindfulness Online	1	comparative	1	104	58
42	eCare for Moods	1	comparative	1	103	100
45	moodManager	2	comparative	1	102	89
56	APT Johansson	1	comparative	1	100	100
114	PopTherapy	4	comparative	1	95	33
75	Alavi Unnamed	1	comparative	1	93	84
99	SUBGAP	1	comparative	1	92	88
19	iCBT-MDD	1	comparative	1	91	72
126	Killen Unnamed	1	comparative	1	88	87
28	Hollandare Unnamed	1	comparative	1	84	77
101	Ly Unnamed Mindfulness	1	comparative	1	81	72
26	Depressionshjalpen	1	comparative	1	80	78
50	Hoorelbeke Unnamed	1	comparative	1	68	67
128	Emotion Diary	1	comparative	1	68	44
69	Feeling Better	3	comparative	1	66	53
5	Avanti	1	comparative	1	63	52
17	Wagner Unnamed	1	comparative	1	62	53
103	Bond Unnamed	1	comparative	1	62	NA
131	OLeary Unnamed	2	comparative	1	61	35

123	CBM Browning	2	comparative	1	61	NA
127	Dobbin Unnamed	1	comparative	1	58	49
86	Calkins Unnamed	1	comparative	1	56	48
38	CBM Peters	1	comparative	1	54	52
57	Agyapong Unnamed	1	comparative	1	54	50
25	Ruwaard Unnamed	1	comparative	1	54	49
100	UPLIFT	1	comparative	1	53	NA
121	CBM Beevers	1	comparative	1	52	44
68	Buhrman Unnamed	1	comparative	1	52	43
9	DAHLIA	1	comparative	1	49	42
48	Strom Unnamed	1	comparative	1	48	48
125	Eisma Unnamed	2	comparative	1	47	36
43	improvehealth.eu	1	comparative	1	46	22
88	Cognitive Therapy: A Multimedia Learning Program	2	comparative	1	45	40
130	CBM Mogoase	1	comparative	1	42	41
120	Kraft Unnamed	1	comparative	1	41	35
89	Interactive Computer-Assisted Psycho-Education System	1	comparative	1	32	32
91	Cognitive Remediation	1	comparative	1	28	21
95	Help4Mood	2	comparative	1	28	21
112	OLDIT	2	comparative	1	24	NA
102	EVO	1	comparative	1	22	22
80	ePST	2	comparative	1	14	13
35	STREAM	1	comparative	1	NA	NA

Table 2. Systems evaluated in non-comparative trials ranked by evidence base.

System Key	*Name of System*	*Number of Versions*	*Evaluation Quality*	*Number of Evaluations*	*Number of Participants Pre*	*Number of Participants Post*
10	The Wellbeing Course	18	non-comp.	9	1131	979
15	Beating the Blues	5	non-comp.	7	584	361
16	Sadness Program	13	non-comp.	5	2198	1090
13	MoodGYM	15	non-comp.	4	80837	22496
66	Janevic Unnamed	4	non-comp.	4	653	375
24	Andersson Unnamed	9	non-comp.	3	72	57
64	ADep	2	non-comp.	2	16584	646
27	Space from Depression	1	non-comp.	2	721	334
18	Smiling is Fun	5	non-comp.	2	101	85
113	Daybuilder	2	non-comp.	2	38	4
94	DCAT ATA	1	non-comp.	1	442	NA
73	Leykin Unnamed	1	non-comp.	1	309	109
59	Pfeiffer Unnamed	1	non-comp.	1	190	66
110	Kawai Unnamed	1	non-comp.	1	168	126
60	MOSS App	1	non-comp.	1	126	12
49	Stress Gym	1	non-comp.	1	110	95
137	Hetrick Unnamed	1	non-comp.	1	101	55
84	Blues Begone	1	non-comp.	1	100	58
88	Cognitive Therapy: A Multimedia Learning Program	2	non-comp.	1	96	75
61	myCompass	1	non-comp.	1	90	49

System Key	*Name of System*	*Number of Versions*	*Evaluation Quality*	*Number of Evaluations*	*Number of Participants Pre*	*Number of Participants Post*
77	Hadjistavropoulos Unnamed	1	non-comp.	1	80	41
67	Ahmedani Unnamed	1	non-comp.	1	75	64
52	LINKS	1	non-comp.	1	52	24
99	SUBGAP	1	non-comp.	1	44	33
32	Alles onder controle	8	non-comp.	1	44	23
12	Rebound	1	non-comp.	1	42	39
21	Creating Opportunities for Personal Empowerment	2	non-comp.	1	39	31
136	ASCENSO	1	non-comp.	1	35	23
8	HIV Tides	1	non-comp.	1	32	NA
80	ePST	2	non-comp.	1	29	23
55	Reframe IT	1	non-comp.	1	27	21
105	MindBalance	1	non-comp.	1	25	18
71	MyStrength	1	non-comp.	1	24	20
134	Overcoming Depression	1	non-comp.	1	22	15
45	moodManager	2	non-comp.	1	21	19
107	Moodscope	1	non-comp.	1	20	16
118	Shamekhi Unnamed	1	non-comp.	1	20	13
97	Falconer Unnamed	1	non-comp.	1	18	15
22	Mansson Unnamed	1	non-comp.	1	15	15
79	Building a Meaningful Life through Behavioral Activation	1	non-comp.	1	15	8
29	van Voorhees Unnamed	1	non-comp.	1	14	8
58	Aguilera Unnamed	1	non-comp.	1	12	10
41	Bae Unnamed	1	non-comp.	1	10	10

System Key	*Name of System*	*Number of Versions*	*Evaluation Quality*	*Number of Evaluations*	*Number of Participants Pre*	*Number of Participants Post*
69	Feeling Better	3	non-comp.	1	10	10
122	Both Unnamed	1	non-comp.	1	9	9
44	Schueller Unnamed	1	non-comp.	1	9	9
96	Health Buddy	1	non-comp.	1	9	9
111	Minddistrict	1	non-comp.	1	9	7
106	T2 Mood Tracker	1	non-comp.	1	8	8
36	CBM Blackwell	6	non-comp.	1	8	7
62	Mobilyze	1	non-comp.	1	8	7
135	Butler system	1	non-comp.	1	4	4
95	Help4Mood	2	non-comp.	1	2	2
70	Mindfulness Online	1	non-comp.	1	NA	273
