# Supplementary material for: Technological State of the Art of Electronic Mental Health Interventions for Major Depressive Disorder: Systematic Literature Review
Source: J Med Internet Res. 2020 Jan 20;22(1):e12599. doi: 10.2196/12599 (PMC6997926; doi:10.2196/12599)
Supplement: Multimedia Appendix 8 [file jmir_v22i1e12599_app8.pdf]

eHDTS score	General system description
**0**	System has no functions delivered by means of technology. Technology might, however, serve as a medium between counsellor and patient, as is the case in telehealth. Additionally, technology might be used to gather data about the user, not for use in the intervention but for the purposes of conducting the study, e.g. e-mails being sent to collect pre- and post-measurements.
**1**	System delivers functions in an informational fashion. For example, a typical CBT intervention might include eight modules of psychoeducation, exercise instructions, and links to additional resources. This is presented on a website and each week of the intervention a new module is made available. The user can click through the different modules but is not prompted to interact with the intervention in any other way. Applying the insights from the educational texts in real life is left to the user. Human guidance is integrated in the same informational manner, e.g. by showing (fictional) video vignettes of other users or similar patients or using prototypical characters. Data might be collected concerning the progress of the user, such as weekly questionnaires or which module they are on and this is then forwarded to a counsellor who might get in touch with the user.
**2**	System functions are interactive but neither responsive nor intelligent. In the case of the core modules, this might mean that the user is prompted to enter information into the system, such as to describe their automatic thoughts or to create an activity schedule. The system, however, does not process this information to any extent beyond simply saving it for the user to see back. When the system collects monitoring data, it presents this back to the user without offering an interpretation, e.g. it might automatically create a graph of daily depression questionnaire scores. The user might be able to tailor the systems to their own needs or preferences by adapting settings, such as what gets displayed on their landing page. New modules might be triggered according to how frequently the user has been interacting with the program. Human guidance may be integrated into the system in an interactive and responsive way, though the responsiveness stems from other humans and not from the system, as is the case in a forum, for example.
**3**	System functions are interactive and responsive to meta-data and might show some intelligence. For example, the core modules might deliver information in accordance with the amount of user activity on the interactive exercises. Some tailoring may be provided in the execution support functionality, but this is based on a static user model created at the outset of the intervention. For example, users might be determined to be male or female initially and the intervention is adapted accordingly. Monitoring data that is collected is processed to draw conclusions for the user and provide feedback. For example, users might see a qualitative interpretation of their depression scale score. The system actively attempts to integrate human support when it detects that the user may need it (e.g. in the case of suicidal ideation).

**4**	System functions are interactive and the system responds in an intelligent way, i.e. it interprets the content of information entered into the system by the user and responds to this content in the form of motivational feedback or changes to the intervention strategy. Additionally, the system tries to meet the user where they are at, for example, by integrating technology they already use (e.g. Google calendar, Facebook feeds, To-Do-List software, physical activity apps). The system dynamically updates its user model based on new monitoring data or information from the user provided through exercises or quizzes and adapts accordingly. Similarly, the system might detect which people are appropriate to contact and with which information given a specific user state. For example, the system might contact a family member if low mood is detected, it contacts a professional in case of suicidal ideation, and it refers the user to an appropriate support forum thread for a more specific problem they just described.
