# Supplementary material for: Technological State of the Art of Electronic Mental Health Interventions for Major Depressive Disorder: Systematic Literature Review
Source: J Med Internet Res. 2020 Jan 20;22(1):e12599. doi: 10.2196/12599 (PMC6997926; doi:10.2196/12599)
Supplement: Multimedia Appendix 9 [file jmir_v22i1e12599_app9.pdf]

**Deciles**	**eHDTS**	**eHDTS**$_w$
10	1.00	0.10
20	1.09	0.22
30	1.25	0.32
40	1.33	0.39
50	1.50	0.45
60	1.67	0.55
70	1.89	0.69
80	2.06	0.82
90	2.53	1.11
100	3.70	2.31
